# Templated‐Construction of Hollow MoS_2_ Architectures with Improved Photoresponses

**DOI:** 10.1002/advs.202002444

**Published:** 2020-10-15

**Authors:** Chao Gao, Yingdong Han, Kun Zhang, Tian Wei, Zhang Jiang, Yang Wei, Lisha Yin, Fabio Piccinelli, Cheng Yao, Xiaoji Xie, Marco Bettinelli, Ling Huang

**Affiliations:** ^1^ Institute of Advanced Materials (IAM) Jiangsu National Synergetic Innovation Center for Advanced Materials (SICAM) Nanjing Tech University 30 South Puzhu Road Nanjing 211816 China; ^2^ School of Inspection and Testing Certification Changzhou Vocational Institute of Engineering Changzhou 213164 China; ^3^ School of Precision Instruments and Optoelectronics Engineering Tianjin University Tianjin 300072 China; ^4^ Luminescent Materials Laboratory Department of Biotechnology University of Verona Verona 37134 Italy; ^5^ School of Chemistry and Molecular Engineering Nanjing Tech University 30 South Puzhu Road Nanjing 211816 China

**Keywords:** energy transfer, hollow MoS_2_, near‐infrared, template, upconversion luminescence

## Abstract

Despite the outstanding optoelectronic properties of MoS_2_ and its analogues, synthesis of such materials with desired features including fewer layers, arbitrary hollow structures, and particularly specifically customized morphologies, via inorganic reactions has always been challenging. Herein, using predesigned lanthanide‐doped upconversion luminescent materials (e.g., NaYF_4_:Ln) as templates, arbitrary MoS_2_ hollow structures with precisely defined morphologies, widely variable dimensions, and very small shell thickness (≈2.5 nm) are readily constructed. Most importantly, integration of the near‐infrared‐responsive template significantly improves the photoresponse of up to 600 fold in device made of NaYF_4_:Yb/Er@MoS_2_ compared with that of MoS_2_ nanosheets under 980 nm laser illumination. Multichannel optoelectronic device is further fabricated by simply changing luminescent ions in the template, e.g., NaYF_4_:Er@MoS_2_, operating at 1532 nm light excitation with a 276‐fold photoresponse enhancement. The simple chemistry, easy operation, high reliability, variable morphologies, and wide universality represent the most important advantages of this novel strategy that has not been accessed before.

## Graphical abstract

A facile template‐based strategy for reliable construction of arbitrary MoS_2_ hollow architectures with widely varied morphologies and dimensions is reported. An up to 600‐fold improvement of photoresponse to 980 nm light illumination is obtained in the representative optoelectronic device made of NaYF_4_:Yb/Er@MoS_2_ composite, compared with that of MoS_2_ nanosheets.

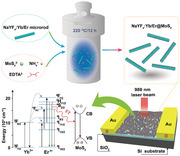

As a shining star among two‐dimensional transition metal dichalcogenides (TMDs), MoS_2_ and its composites have aroused impressive research enthusiasms due to their outstanding physical and chemical properties, such as large surface area, excellent electric conductivity, adjustable band gap, and tailorable density of active sites. Such superior features have imparted them great potentials in many applications including host materials for energy storage in metal ion batteries and super capacitors, catalysts for hydrogen evolution reactions, as well as devices for gas sensors, flash memories, and photodetectors.^[^ ^1^, ^2^, ^3^, ^4^, ^5^, ^6^, ^7^, ^8^, ^9^, ^10^ ^]^

In virtue of its sandwich‐like structure coupled by van der Waals forces and the in‐plan S—Mo—S covalent bonding, numerous efforts have been invested aiming to exfoliate bulk MoS_2_ into single or few‐layered sheets to satisfy different requirements.^[^ ^11^, ^12^ ^]^ However, one of the serious challenges is that the exfoliated nanosheets are prone to reaggregate or restack when extracted from solution, causing even massier piling, such as twisted, folded, or broken pieces and resulting in significantly deteriorated performance.^[^ ^13^, ^14^, ^15^ ^]^ Thus, MoS_2_ architectures with large surface area, particularly those hollow structures with precisely designable and retainable configurations are highly demanded.^[^ ^16^, ^17^, ^18^ ^]^ For example, Li et al. synthesized ≈100 nm thick hollow microcube framework of MoS_2_ with high specific capacity and large electrolyte/electrode contact for sodium storage;^[^ ^16^ ^]^ Zhuo et al. constructed hierarchical MoS_2_ nanotubes with a layer thickness of ≈50 nm through anion exchange and improved photocurrent was obtained;^[^ ^17^ ^]^ Yu et al. fabricated cubic hollow MoS_2_ structures with a thickness of ≈20 nm, aiming at maximizing the exposed edge sites to promote its catalytic activity.^[^ ^18^ ^]^ Nonetheless, those architectures are still tens or hundreds of times thicker than the value (6.5 Å) characteristic of a single layer MoS_2_,^[^ ^8^ ^]^ and there remains a large room for further improving the ratio of exposed surface area to the mass of MoS_2_. Thus, hollow structures with even thinner shells possessing large surface‐to‐volume ratio, high percentage of exposed atoms, customizable morphologies, and thus better performance in electrochemical catalysis and enhanced sensitivities in optoelectronic devices are highly desired.

Meanwhile, although lanthanide‐doped upconversion luminescent materials have been thoroughly studied for their unique optical response under near‐infrared (NIR) illumination,^[^ ^19^, ^20^, ^21^, ^22^ ^]^ the simple inorganic chemistry of lanthanide fluorides for being used as template for MoS_2_ hollow structure synthesis remains untouched. Specifically, templates with widely designable morphologies and dimensions can be easily synthesized, and they can also be neatly removed by just soaking in acidic solution.^[^ ^23^ ^]^ Thus, a series of arbitrary MoS_2_ hollow structures with shells as thin as ≈2.5 nm and dimensions ranging from as small as 35 nm to as large as several micrometers, can all be reliably constructed. More intriguingly, upconversion luminescence (UCL) at the wavelength of 540 and 654 nm generated by doped Yb^3+^/Er^3+^ in the template under NIR light excitation (980 nm), falls completely within the absorption range of MoS_2_ (350–950 nm), which facilitates efficient resonance energy transfer (ET) and significantly improves optoelectronic responses. Following the same design principle, it has also been possible to fabricate photodetectors working at other wavelength (1532 nm) by simply changing the luminescent ions of the template, demonstrating great potential for interdisciplinary applications.

As illustrated in **Scheme** **1**, using the most thoroughly studied NaYF_4_:Yb/Er (18/2 mol%) as a representative template, composites of NaYF_4_:Yb/Er@MoS_2_ possessing expected morphologies and dimensions are routinely obtained by mixing the presynthesized template with precursors for MoS_2_ thin layer growth, and then submitting the resulting solution to a hydrothermal reaction. Corresponding hollow structures are left after removal of NaYF_4_:Yb/Er template via a facile washing of the composite using diluted aqueous solution of HCl.

**Scheme 1:**
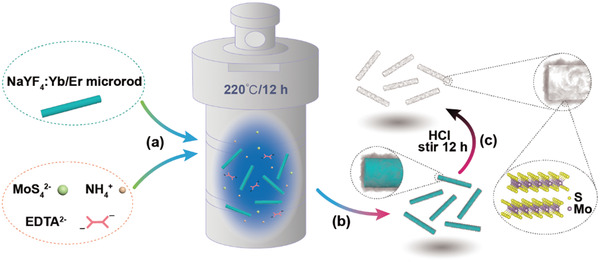
Schematic illustration of the synthetic procedures of hollow MoS_2_ architectures. a) Preparation of reacting ingredients inside an autoclave, b) synthesized composites, and c) microtubes obtained after removal of template.

**Figure** **1**a shows transmission electron microscopy (TEM) image of the as‐synthesized MoS_2_ nanosheets with typical interlayer distance of 0.658 nm. The scanning electron microscopy (SEM) image in Figure 1b indicates high uniformity of NaYF_4_:Yb/Er microrods used as template for hollow MoS_2_ microtube construction. X‐ray diffraction (XRD) patterns of NaYF_4_:Yb/Er@MoS_2_ matches well with those of standard XRD data of each individual component (Figure 1c). Both the thin layers of MoS_2_ and the encapsulated NaYF_4_:Yb/Er microrods are easily seen in TEM image (Figure 1d), and the interface is clearly discernable in the zoomed‐in TEM image (Figure 1e). The 0.710 nm interlayer distance in high‐resolution TEM (HRTEM) image (Figure 1f) matches with that of MoS_2_. This is also highly consistent with the elemental mapping results where a sharp edge between the distribution of Y, F, and S, Mo elements at one end of the microrod is manifested (Figure 1g; and Figure S1, Supporting Information). The microtubes are then obtained after removal of NaYF_4_:Yb/Er template and the structural details of MoS_2_ particularly the 0.713 nm characteristic interlayer distance are shown in Figure 1h–j. Compared with the value of 0.658 nm in pristine MoS_2_, the increased interlayer distance is likely caused by the nonplanar stacking of individual MoS_2_ layers. It is worth emphasizing that the morphology of microtubes was well retained and almost identical to that of template. We also affirmed that the hexagonal‐phase structure was maintained in both MoS_2_ microtubes and MoS_2_ nanosheets as indicated in the XRD data (Figure S2, Supporting Information). It should be noted that the peak intensity attributed to the (002) lattice plane becomes weaker in MoS_2_ microtubes than that in MoS_2_ nanosheets. This suggests that MoS_2_ microtubes might have fewer layers, which is also consistent with the high transparency seen in Figure 1h.^[^ ^24^, ^25^ ^]^

**Figure 1:**
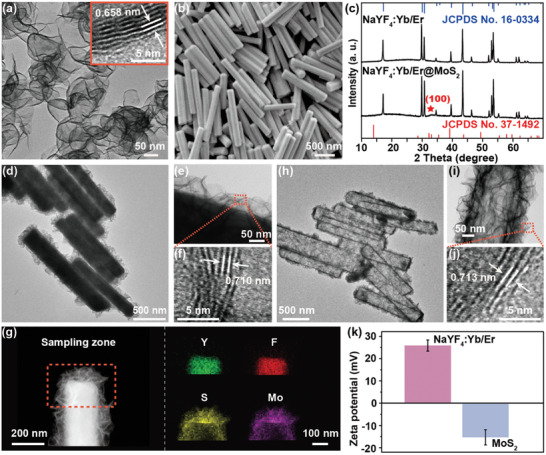
a) TEM image of as‐synthesized MoS_2_ nanosheets. b) SEM image of as‐synthesized NaYF_4_:Yb/Er template. c) XRD patterns of NaYF_4_:Yb/Er@MoS_2_ composite and NaYF_4_:Yb/Er microrods. The top and bottom patterns are standard XRD patterns of NaYF_4_:Yb/Er and MoS_2_, respectively. d) TEM image of NaYF_4_:Yb/Er@MoS_2_ composite. e) Magnified TEM image of sample in (d). f) HRTEM image of MoS_2_ layer in NaYF_4_:Yb/Er@MoS_2_ composite shown in (e). g) Elemental mapping of the NaYF_4_:Yb/Er@MoS_2_ composite showing sharp distribution of Y, F and S, Mo. h) TEM image of MoS_2_ microtubes after removal of NaYF_4_:Yb/Er template. i) Magnified TEM image of sample in (h). j) HRTEM image of the edge of MoS_2_ microtube shown in (i). k) Zeta potential profile of ligand‐free NaYF_4_:Yb/Er microrods and as‐synthesized MoS_2_ nanosheets shown in (a).

The Raman spectrum of MoS_2_ microtubes has two characteristic peaks at 380.4 and 403.9 cm^−1^ (Figure S3, Supporting Information), which correlates to the in‐plane (E^1^_2g_) and out‐of‐plane vibration (A_1g_) mode of hexagonal MoS_2_, respectively. The frequency difference between the E^1^_2g_ and A_1g_ Raman modes is ≈23.5 cm^−1^, which suggests very few layers of the MoS_2_ microtube and agrees with the TEM results.^[^ ^26^ ^]^ We also performed energy dispersive spectroscopy (EDS) characterization of the as‐synthesized hollow MoS_2_ microtubes (Figure S4, Supporting Information), where no signals ascribable to Na^+^, F^−^, or rare earth ions (RE^3+^) can be detected. This indicates that the template was completely removed by soaking in aqueous HCl solution followed by a thorough wash.

Zeta potential measurement shows positive charge in NaYF_4_:Yb/Er template, which originates from the exposed RE^3+^ when ligand molecules are stripped off from the microrod surface (Figure 1k).^[^ ^27^ ^]^ The negative charge on MoS_2_ makes it prone to combine to the surface of NaYF_4_:Yb/Er through electrostatic attractions, enabling natural growth of MoS_2_ thin layers. However, since S^2−^ is a soft base while RE^3+^ is a hard acid according to the theory of hard and soft acids and bases formulated by Pearson,^[^ ^28^, ^29^ ^]^ their combination is so weak that only small and isolated pieces of MoS_2_ can bind randomly on NaYF_4_:Yb/Er surface (Figure S5, Supporting Information), resulting in poor‐quality of broken MoS_2_ hollow structures after template removal (Figure S6, Supporting Information).

To solve this problem, a surfactant molecule with stronger chelating capabilities to RE^3+^, i.e., a harder base, disodium salt of ethylenediamine tetraacetic acid (EDTA‐2Na), was intentionally added into the reaction system. We expect that EDTA^2−^ will bind firmly to RE^3+^ on the template surface and meanwhile help to fix MoS_2_ precursors (Mo^6+^ ions) from solution and further regulate the growth of MoS_2_ thin layers with improved quality,^[^ ^30^, ^31^ ^]^ when reaction conditions such as precursor concentration, reaction temperature, and growth time, are optimized. This also explains why the growth of MoS_2_ thin layers follows so closely to the contour of the template (Figure 1h,i). Moreover, the very thin layer of hollow MoS_2_ may favorite higher density of active sites or electronic sensitivities than thicker analogues when used for photo‐ and/or electrochemical catalysis or optoelectronics.

The interactions between template and shell in NaYF_4_:Yb/Er@MoS_2_ composite were further studied using X‐ray photoelectron spectroscopy (XPS). The binding energies of Mo 3d_3/2_ and Mo 3d_5/2_ in pure MoS_2_ nanosheets located at 232.38 and 229.18 eV shifted to 231.74 and 228.34 eV in NaYF_4_:Yb/Er@MoS_2_ (**Figure** **2**a; and Figure S7, Supporting Information), while those of S 2p_1/2_ and S 2p_3/2_ observed at 163.20 and 162.01 eV in pure MoS_2_ nanosheets shifted to 162.74 and 161.54 eV (Figures S7 and S8, Supporting Information), respectively. This behavior is presumably caused by the increased electron cloud density of the outer orbitals of Mo^4+^ and S^2−^ ions, which suggests the presence of a strong electrostatic interaction between MoS_2_ and NaYF_4_:Yb/Er in the composite. More importantly, the respective binding energies for MoS_2_ microtubes fell back to the values similar to those of pure MoS_2_ nanosheets when NaYF_4_:Yb/Er templates were removed (Figure S7, Supporting Information), which reversely confirms the existence of electrostatic interaction.

**Figure 2:**
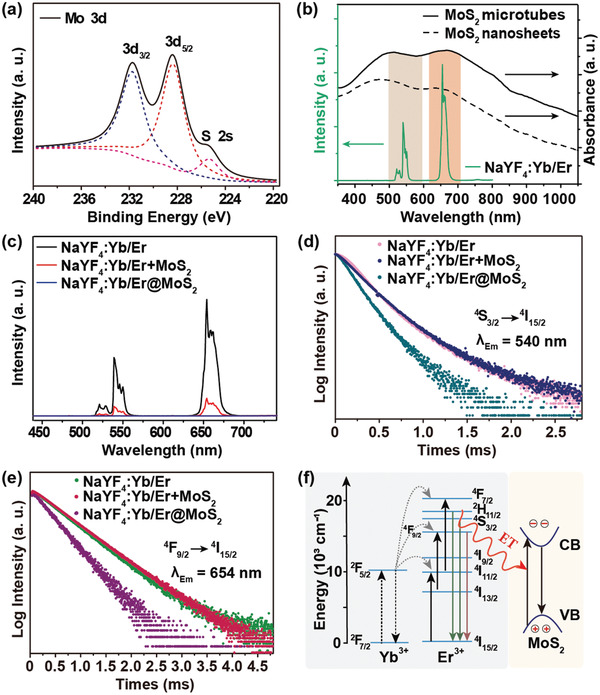
a) XPS of Mo 3d orbitals in NaYF_4_:Yb/Er@MoS_2_ composite. b) UV–Vis–NIR absorption spectra of MoS_2_ microtubes and nanosheets (right) and UCL spectrum of NaYF_4_:Yb/Er (left) under 980 nm laser excitation. c) UCL spectra of NaYF_4_:Yb/Er, NaYF_4_:Yb/Er+MoS_2_, and NaYF_4_:Yb/Er@MoS_2_ composite under 980 nm laser excitation. Lifetime decay curves of NaYF_4_:Yb/Er, NaYF_4_:Yb/Er+MoS_2_, and NaYF_4_:Yb/Er@MoS_2_ composite at emission wavelength of d) 540 and e) 654 nm. f) Proposed ET pathways from NaYF_4_:Yb/Er to MoS_2_ inside the NaYF_4_:Yb/Er@MoS_2_ composite.

The complete spectral overlap between the UV–Visible–NIR absorption of MoS_2_ and the UCL emissions of NaYF_4_:Yb/Er under 980 nm laser excitation (Figure 2b; and Figure S9, Supporting Information) allows efficient nonradiative ET between the two components, which is reflected by the significantly decreased UCL intensity of NaYF_4_:Yb/Er@MoS_2_ compared with that of pure NaYF_4_:Yb/Er (Figure 2c). As a control experiment, the UCL of NaYF_4_:Yb/Er in its physical mixture with MoS_2_ is still observable, although greatly weakened (Figure 2c), which might be caused by the UCL reabsorption by MoS_2_ as well as light scattering from small pieces of MoS_2_. Accordingly, shortening of emission lifetimes at 540 and 654 nm from 243 and 494 µs in NaYF_4_:Yb/Er to 182 and 416 µs in the composite, respectively, suggests the obvious ET from NaYF_4_:Yb/Er to MoS_2_, while no prominent lifetime change was detected in their physical mixture (Figure 2d,e). The efficiency of nonradiative ET process is estimated to be around 25% for ^4^S_3/2_ level and 15% for ^4^F_9/2_ level.^[^ ^32^ ^]^ Figure 2f depicts the nonradiative ET pathways where the energy responsible for UCL emissions is transferred directly to the closely bound MoS_2_ thin layers.^[^ ^33^ ^]^ Moreover, the 1.276 eV bandgap of MoS_2_ thin layers (Figure S10, Supporting Information) matches with both emissions at 540 and 654 nm of the microrods, which shall be responsible for the lifetime decrease (Figure 2d,e).

To showcase the improved NIR‐response of 3D MoS_2_ hollow architectures brought by the NIR‐responsive template,^[^ ^34^ ^]^ a photodetector was fabricated (**Figure** **3**a) using NaYF_4_:Yb/Er@MoS_2_ composite shown in Figure 1d. The optical image of the photodetector is displayed in Figure S11a (Supporting Information), of which the channel length and width is 100 and 1000 µm, respectively. The thickness of NaYF_4_:Yb/Er@MoS_2_ film is about 1 µm as shown in Figure S11b (Supporting Information). Current–voltage (*I–V*) measurements under dark and illumination conditions give linear characteristics at low voltage (Figure 3b), demonstrating a typical ohmic contact between the electrodes and the channel materials,^[^ ^35^ ^]^ which makes bias voltage applied on the active channel rather than on the contact interface between electrode and channel material, and ensures high electrical responsivity. Comparison of Figure 3b and Figure S12 (Supporting Information) indicates that the dark current of the composite is much larger than that of pure MoS_2_ nanosheets, suggesting higher electrical conductivity of the composite. The negative photoresponse in pure MoS_2_ nanosheets (Figure S12, Supporting Information) is likely due to the competitive effects between photogenerated charger carriers and photothermal effect.^[^ ^36^, ^37^, ^38^ ^]^ It should be noted that *I‐V* curves of photodetector made of pure MoS_2_ nanosheets showed Schottky‐contacted characteristics (Figure S12, Supporting Information), while that of NaYF_4_:Yb/Er@MoS_2_ composite showed ohmic‐contact features (Figure 3b), which was likely due to the 3D structure of NaYF_4_:Yb/Er@MoS_2_ compared to 2D MoS_2_ nanosheets. Because the special 3D framework makes MoS_2_ contacted with metal electrode mostly in the way of “edge‐contact,” while MoS_2_ nanosheets mainly in the way of “top‐contact” and the former style was recognized helpful for reducing contact resistance and improving electrical properties.^[^ ^39^, ^40^, ^41^ ^]^

**Figure 3:**
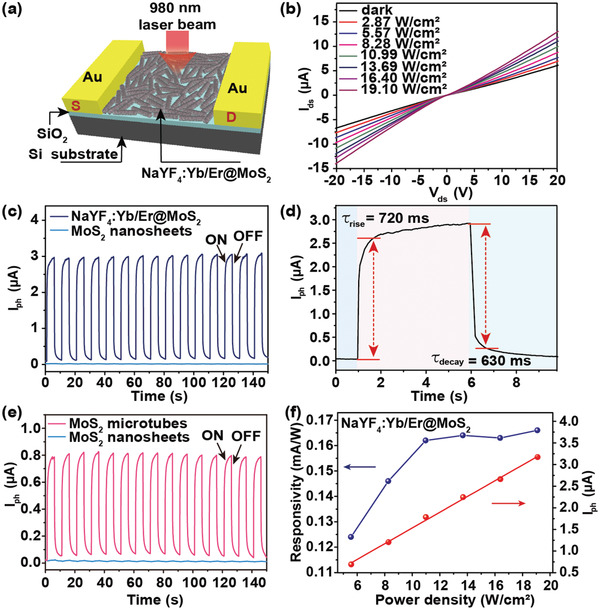
a) Schematic illustration of the optoelectronic device fabricated using NaYF_4_:Yb/Er@MoS_2_ composite with channel length of 100 µm and width of 1000 µm. b) Dependence of the *I–V* curves on illumination power density of the device in (a), under 980 nm laser excitation. c) Temporal photocurrent responses of devices made of NaYF_4_:Yb/Er@MoS_2_ and MoS_2_ nanosheets, respectively. d) Time‐resolved photocurrent indicating the rise and decay time after 980 nm laser switching ON or OFF. e) Temporal photocurrent responses of devices made of MoS_2_ microtubes and MoS_2_ nanosheets, respectively. f) Photocurrent and responsivity of NaYF_4_:Yb/Er@MoS_2_ device as a function of illumination power density at 980 nm and voltage at 10 V.

As an important parameter for NaYF_4_:Yb/Er@MoS_2_ photodetector, the temporal response was recorded under excitation of a pulsed laser with time interval of 10 s. The highly repeatable and stable photocurrent under 980 nm laser irradiation was evidently enhanced in NaYF_4_:Yb/Er@MoS_2_ composite by 600 folds compared with that of pure MoS_2_ nanosheets at the illumination power density of 19.10 W cm^−2^ and voltage of 10 V (Figure 3c), which displays an excellent light switching functionality of this device. The time‐resolved photocurrent rises accordingly with increased excitation power density or applied voltage (Figures S13 and S14, Supporting Information). The switching time for the photocurrent rise, defined as the time for the rise of the output signal from 0% to 90% of the maximal output value,^[^ ^42^ ^]^ is 0.72 s for NaYF_4_:Yb/Er@MoS_2_ photodetector (Figure 3d), while the one for decay, defined as the time for the decrease from the maximal out value to 10%, is 0.63 s (Figure 3d).

Only limited photocurrent can be generated in pure MoS_2_ nanosheets under NIR (980 nm) excitation due to the comparatively large bandgap (1.276 V). However, when the template NaYF_4_:Yb/Er was integrated in the photodetector, the synergetic effect derived from the 3D architecture of MoS_2_ microtubes and non‐radiative ET from NaYF_4_:Yb/Er greatly promotes the NIR photoresponse of MoS_2_. As a contrast, a 160‐fold enhanced photoresponse was still obtained in photodetector made of MoS_2_ microtubes (Figure 3e), which could be similarly attributed to the increased electric conductivity of the 3D structure compared with that of MoS_2_ nanosheets.

MoS_2_ can directly absorb illuminating photons above its band gap and generate charge carriers, and the photocurrent increases with the light intensity following the power‐law of^[^ ^43^ ^]^

(1) $$\begin{array}{c} I_{\mathrm{ph}}\propto I^{m} \end{array}$$

in which *I*_ph_ is the current when light is ON and *I* is the emission intensity of the NIR light source, which can be directly absorbed by MoS_2_. For NaYF_4_:Yb/Er@MoS_2_, *I* should be the intensity of UCL emitted from NaYF_4_:Yb/Er microrods, which is proportional to the excitation laser power density

(2) $$\begin{array}{c} I\propto P_{\mathrm{Ex}}^{n} \end{array}$$

where *n* is defined as the number of photons required for UCL, and *P*_Ex_ is the laser power density. Therefore, it can be figured out that

(3) $$\begin{array}{c} I_{\mathrm{ph}}\propto P_{\mathrm{Ex}}^{mn} \end{array}$$

So for NaYF_4_:Yb/Er@MoS_2_ photodetector, through exponential fit shown in Figure 3f, the calculated *mn* is 1.14. The number of photons for green and red upconversion emissions were obtained by fitting the curve of emission intensity versus excitation power (Figure S15, Supporting Information). Because both green and red emissions can be absorbed by MoS_2_, we here applied the average photon number for green and red upconversion emission, *n* = 1.87. Therefore the calculated value for *m* is 0.61, which falls within the range of 0 < *m* < 1, and suggests the typical semiconductor property involving electron–hole separation, trapping, and recombination.^[^ ^44^ ^]^ Moreover, the NIR response of this photodetector can be further amplified ≈1.8 folds (Figure S16a, Supporting Information) by coupling with Au nanorods at dimensions of 15 nm × 95 nm (Figure S17, Supporting Information), whose localized surface plasmon resonance matches exactly with the excitation wavelength of NaYF_4_:Yb/Er at 980 nm (Figure S16b, Supporting Information). This result, again, proves not only the critical role that the template plays but also the rationality of our design principle.

To further evaluate the photoresponse performance, responsivity (*R*), specific detectivity (D*), and external quantum efficiency (EQE) were evaluated. *R* represents the electric response to excitation light and is defined as *I*_ph_/*AP*_Ex_, where *A* is the effective area of the detector. D* stands for the ability of a photodetector in detecting weak signals, and is defined as *RA* ^1/2^/(2*eI*_dark_)^1/2^, where *e* is electron charge. *EQE* is defined as *hcR/eλ*, where *h* is the Plank constant, *c* is the light velocity.^[^ ^35^, ^43^ ^]^ Therefore we can deduce that the *R* of NaYF_4_:Yb/Er@MoS_2_ photodetector kept increasing and reached steady state (Figure 3f) with laser power increasing. Moreover, compared with MoS_2_ nanosheet photodetector, *R*, D*, and *EQE* are increased from 0.268 × 10^−3^ mA W^−1^, 9.3 × 10^4^ Jones and 0.34 × 10^−4^% to 166 × 10^−3^ mAW^−1^, 560 × 10^4^ Jones and 210 × 10^−4^%, respectively. Table S1 (Supporting Information) also summarized previously reported results where we can find that our work shows similar or even better performance. However, we believe that after further optimization of the material design parameters, fabrication steps, and working conditions, devices made of NaYF_4_:Yb/Er@MoS_2_ composite shall exhibit even better NIR photoelectric detectivity.

Taking advantage of the multiple absorption capability of Er^3+^, the working wavelength of such photodetector can be readily tuned to another range. For example, in the photodetector made of NaYF_4_:Er@MoS_2_ where Er^3+^ is responsive to 1532 nm laser illumination (Figure S18, Supporting Information), a 276‐fold photoresponse enhancement was obtained compared with that of pure MoS_2_ nanosheets (Figure S19, Supporting Information). Similarly, this was also due to the ET from Er^3+^ in the template to the MoS_2_ shell (Figure S20, Supporting Information) according to the lifetime changes of the luminescence emissions at 540 and 654 nm (Figure S21, Supporting Information), with efficiency of 22% and 14%, respectively. The photodetector made of MoS_2_ microtubes gave a 155‐fold improvement (Figure S19, Supporting Information), which is almost identical to that of 160‐fold shown in Figure 3e and confirms reliable quality of the MoS_2_ microtubes though different templates were used, i.e., NaYF_4_:Yb/Er versus NaYF_4_:Er. It needs to be pointed out that although under the same laser illumination power density, it is reasonable to see smaller photocurrent in NaYF_4_:Er@MoS_2_ photodetector under 1532 nm laser illumination (Figure S19, Supporting Information) compared with that of NaYF_4_:Yb/Er@MoS_2_ excited with 980 nm laser (Figure 3c) because: 1) Er^3+^ has smaller absorption cross section (1.7 × 10^−21^ cm^2^ at 1532 nm) in NaYF_4_:Er than that of Yb^3+^ (9.11 × 10^−21^ cm^−2^ at 980 nm) in NaYF_4_:Yb/Er,^[^ ^33^ ^]^ and 2) the relatively low upconversion efficiency of Er^3+^ in NaYF_4_:Er, which is a 3‐photon upconversion process compared with a 2‐photon process in NaYF_4_:Yb/Er.

Due to the very close ionic radii and highly similar chemical properties of lanthanide ions, Gd^3+^ was intentionally codoped for more sophisticated dimension and morphology tuning of the templates. Following the same recipe, a wide variety of MoS_2_ hollow architectures could then be readily obtained when NaYF_4_:Ln (Ln = Yb/Er, 18/2 mol% for nanoparticles, nanoplates, hexagonal prisms, and super microrods; Ln = Yb/Er/Gd, 18/2/30 mol% for nanorods) with pre‐designed dimensions and morphologies were used as templates (**Figure** **4**), such as nanovesicles as small as 35 nm (Figure 4a,f,k), hollow hexagonal prisms with height varying from 50 (Figure 4b,g,l) to 240 nm (Figure 4c,h,m), nanotubes (Figure 4d,i,n), and super microtubes (Figure 4e,j,o), respectively. Detailed analysis of TEM images of the nanovesicles indicates only ≈2.5 nm average thickness of the MoS_2_ shell (Figure S22, Supporting Information), which is so far the smallest value ever reported. Moreover, it is worth emphasizing that not only the thin layer grows very uniformly along the templates, the structural details such as the 120° internal angle (Figure 4l,m) of the hollow hexagonal prism, and the sharp end of the super microtube (Figure 4o) derived from respective templates, were also perfectly retained in the hollow architectures. This, on the other hand, reflects perfect control for high quality hollow architecture synthesis originated from the sufficient affinity between MoS_2_ and NaYF_4_:Ln, and introduction of Gd^3+^ does not affect the quality of the composite or template removal. Such widely designable hollow architectures may possess great potentials in drug delivery, energy storage, thin film separation, as well as electrochemical and/or photocatalysis.

**Figure 4:**
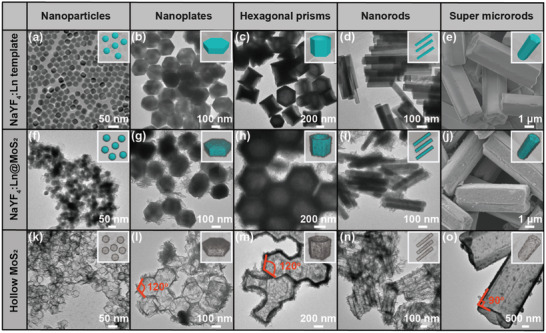
TEM images of NaYF_4_:Ln with morphologies of a) nanoparticle, b) nanoplate, c) hexagonal prism, d) nanorod, and e) super microrod, which were used as templates for growing of corresponding composites showing in (f–j). k–o) are TEM images of the corresponding hollow MoS_2_ architectures after template removal.

In conclusion, we have developed a facile and reliable template‐based strategy for arbitrary construction of hollow MoS_2_ architectures with dimensions ranging from as small as ≈35 nm to up to as large as ≈10 µm, and morphologies ranging from nanoscaled vesicles to microsized tubes. This is the first time that easy fabrication of various MoS_2_ hollow structures with shells as thin as ≈2.5 nm was ever accessed. As an added value, the lanthanide template is able to upconvert the NIR excitation energy and transfer efficiently to the MoS_2_ shell, which can be further exploited for the development of multichannel NIR photodetectors. Moreover, the strategy developed here should inspire further constructions of other TMD hollow architectures with outstanding performances not only in optoelectronics, but also potentially in energy storage and photo‐/electrochemical catalysis, which are ongoing projects in our lab.

## Conflict of Interest

The authors declare no conflict of interest.

## Supporting information

Supporting Information
