# Supplementary material for: Templated‐Construction of Hollow MoS2 Architectures with Improved Photoresponses
Source: Adv Sci (Weinh). 2020 Oct 15;7(22):2002444. doi: 10.1002/advs.202002444 (PMC7675057; doi:10.1002/advs.202002444)
Supplement: Supplementary file 1 — Supporting Information [file ADVS-7-2002444-s001.pdf]

© 2020 Wiley-VCH GmbH

ADVANCED SCIENCE
Open Access

Supporting Information

for *Adv. Sci.,* DOI: 10.1002/advs.202002444

Templated-Construction of Hollow MoS_2 Architectures with Improved Photoresponses

Chao Gao, Yingdong Han, Kun Zhang, Tian Wei, Zhang Jiang, Yang Wei, Lisha Yin, Fabio Piccinelli, Cheng Yao, Xiaoji Xie, Marco Bettinelli, Ling Huang**

Supporting Information

Templated-Construction of Hollow MoS_2 Architectures with Improved Photoresponses

Chao Gao†, Yingdong Han†, Kun Zhang, Tian Wei, Zhang Jiang, Yang Wei, Lisha Yin, Fabio Piccinelli, Cheng Yao, Xiaoji Xie, Marco Bettinelli, Ling Huang**

Dr. C. Gao, Dr. K. Zhang, T. Wei, Z. Jiang, Y. Wei, Prof. L. Yin, Prof. X. Xie, Prof. L. Huang,
Institute of Advanced Materials (IAM), Jiangsu National Synergetic Innovation Center for Advanced Materials (SICAM), Nanjing Tech University, 30 South Puzhu Road, Nanjing, 211816, China
E-mail: iamxjxie@njtech.edu.cn; iamlhuang@njtech.edu.cn

Dr. C. Gao
School of Inspection and Testing Certification, Changzhou Vocational Institute of Engineering, Changzhou, 213164, China

Dr. Y. Han
School of Precision Instruments and Optoelectronics Engineering, Tianjin University, Tianjin, 300072, China

Prof. C. Yao
School of Chemistry and Molecular Engineering, Nanjing Tech University, 30 South Puzhu Road, Nanjing, 211816, China

Prof. Fabio Piccinelli, Prof. Marco Bettinelli
Luminescent Materials Laboratory, Department of Biotechnology, University of Verona, Verona, 37134, Italy
† These authors contributed equally to this work.

Keywords: template, hollow MoS_2, upconversion luminescence, near-infrared, energy transfer

Experimental Details

Materials

Yttrium (III) nitrate (99.9%), ytterbium (III) nitrate (99.9%), erbium (III) nitrate (99.9%), yttrium (III) chloride (99.9%), ytterbium (III) chloride (99.9%), erbium (III) chloride (99.9%), gadolinium (III) chloride (99.9%), ammonium tetrathiomolybdate ($(NH_4)_2MoS_4$, 99.95%), sodium hydroxide (NaOH, > 98%), ammonium fluoride (NH_4F, > 98%), oleic acid (OA, 90%), 1-octadecene (ODE, 90%), ethylenediamine tetraacetic acid disodium salt (EDTA-2Na, > 99%), ethylenediamine tetraacetic acid (EDTA, > 99.5%) and sodium fluoride (NaF, > 98%) were all purchased from Sigma-Aldrich and used as received.

Characterization

The X-ray powder diffraction patterns were performed on a Rigaku D/max 2550 X-ray diffractometer using Cu Kα radiation ($\lambda = 0.154$ nm). Transmission electron microscope (TEM) images were obtained on a JEM-1400 PLUS transmission electron microscope, operating at an acceleration voltage of 100 kV. Scanning electron microscope (SEM) images and energy dispersive spectroscopy (EDS) were characterized by a JSM-7800F field emission scanning electron microscope (Japan Electron Optics Laboratory Co. Ltd., JEOL) operating at 5 kV. High resolution transmission electron microscopy (HRTEM) and elemental mapping were carried out on a Tecnai G2 F20 field emission transmission electron microscope equipped with an energy-dispersive X-ray system operating at 200 kV. The Raman spectrum was collected from Raman spectrometer (Witec alpha 300M+) having an excitation wavelength of 488 nm. The absorption

spectra were tested on a Shimadzu UV-1750 ultraviolet-visible spectrophotometer. Upconversion luminescence spectra were recorded on a Fluorolog®-3 spectrofluorometer by Horiba using an external 980 nm or 1532 nm laser as excitation source. The optical image was obtained on Olympus bx53 optical microscope. The film thickness was tested on KLA Tencor P-7 Stylus Profiler (USA). The electric measurements were carried out with a Keithley 4200 SCS instrument equipped with infrared light laser. All measurements were taken at room temperature.

Synthesis of $NaYF_4$:Yb/Er or $NaYF_4$:Er microrods

The $NaYF_4$:Yb/Er (18/2 mol%) or $NaYF_4$:Er (10 mol%) microrods were synthesized following a similar hydrothermal method. Typically, 1.5 mL aqueous solution of NaOH (0.3 g; 7.5 mmol), 5 mL of OA and 5 mL of ethanol were mixed and under vigorous stirring for 40 min. Thereafter, an aqueous solution of NH_4F (2 M, 1 mL) was added into the above mixture and stirred for 40 min. Subsequently, 2 mL of aqueous solution containing $RE(NO_3)_3$ (0.2 M, $Y^{3+}/Yb^{3+}/Er^{3+}$ = 80/18/2 mol% or Y^{3+}/Er^{3+} = 90/10 mol%) was added and the solution was kept stirring for 1 h. Finally, the resulting mixture was transferred into a 20 mL Teflon-lined autoclave and heated at 220 °C for 12 h. After cooling down to room temperature, the product was obtained by centrifugation followed with washing in ethanol and water for three times each. The obtained $NaYF_4$:Yb/Er or $NaYF_4$:Er microrods were dispersed in 4 mL of cyclohexane.

Surface ligand-stripping from $NaYF_4$:Yb/Er microrods

The capping ligands on the surface of as-synthesized $NaYF_4$:Yb/Er microrods were removed for templated MoS_2 growth. In a typical procedure, 1 mL cyclohexane

dispersion of $NaYF_4$:Yb/Er microrods (0.1 M) mixed with 800 μL ethanol was centrifuged at 12000 rpm for 1 min. The precipitated product was dissolved with 750 μL of ethanol and 500 μL of HCl (1 M), and was centrifuged at 12000 rpm for 15 min. The precipitated product was added into 750 μL of ethanol and 50 μL of HCl (1 M) and was centrifuged again at 12000 rpm for 15 min. At last the ligand-free microrods were obtained and dispersed in 500 μL deionized water for further use.

Synthesis of MoS_2 nanosheets

$(NH_4)_2MoS_4$ (0.2 mmol) was dissolved into 15 mL distilled water with vigorous stirring to get a homogenous solution. Then the solution was transferred into a 20 mL Teflon-lined autoclave and maintained at 220 °C for 12 h. The final product was washed with distilled water and ethanol, collected by centrifugation, and then dried at 50 °C in vacuum for 12 h.

Synthesis of $NaYF_4$:Yb/Er@MoS_2 composite

The $NaYF_4$:Yb/Er@MoS_2 composite was synthesized by a hydrothermal method. Typically, 8 mL aqueous solutions of EDTA-2Na (0.04 mmol) and NaF (0.2 mmol) were mixed. Then an aqueous solution containing ligand-free $NaYF_4$:Yb/Er microrods (0.2 M, 1 mL) was added into the above mixture under vigorous stirring for 40 min. Thereafter, 6 mL aqueous solution of $(NH_4)_2MoS_4$ (0.2 mmol) was added and the solution was kept stirring for 1 h, which was then transferred into a 20 mL Teflon-lined autoclave and subsequently heated at 220 °C for 12 h. After naturally cooling down to room temperature, the product was collected by centrifugation and washing by distilled water and ethanol. The resulting black product was dried at 50 °C in vacuum for 12 h.

Removal of $NaYF_4$:Yb/Er microrods from $NaYF_4$:Yb/Er@MoS_2 composite

The template of as-synthesized $NaYF_4$:Yb/Er@MoS_2 composite was etched by immersing in a diluted aqueous solution of HCl. In a typical procedure, $NaYF_4$:Yb/Er@MoS_2 composite (30 mg) were added into 12 mL of HCl (1 M) and the solution was kept stirring for 12 h. Finally, the product was obtained by centrifugation and washed by distilled water and ethanol, then dried at 50 °C in vacuum for 12 h.

Synthesis of $NaYF_4$:Yb/Er nanoparticles

$NaYF_4$:Yb/Er (18/2 mol%) nanoparticles were synthesized by a thermal coprecipitation method. The specific steps are as follows: 0.8 mmol of YCl_3, 0.18 mmol of $YbCl_3$, 0.02 mmol of $ErCl_3$, 7.5 mL of OA and 17.5 mL of ODE were added into a 50 mL three-necked flask. The solution was heated to 150 °C for 30 min under N_2 flow and then cooled down to room temperature. 15 mL of methanol solution containing 4 mmol NH_4F and 2.5 mmol NaOH was added to the above reaction, and the temperature was raised to 100 °C for 30 min to remove methanol in the solution. Thereafter, the reaction temperature was raised to 300 °C for 1.5 h in a nitrogen atmosphere. After cooling down, the solution was centrifuged three times with a mixed solution of ethanol and cyclohexane, and the final product was dispersed in 10 mL of cyclohexane.

Synthesis of $NaYF_4$:Yb/Er nanoplates

The $NaYF_4$:Yb/Er (18/2 mol%) nanoplates were synthesized by a hydrothermal method. The synthesis procedure is as follows: 1.6 mL of ethanol, 1.6 mL of OA, and 9 mL of H_2O were added into 1.5 mL aqueous solution of NaOH (2.5 mmol) and the mixture was stirred for 40 min. Then 0.33 mL aqueous solution of NH_4F (2 M) was added into

the above solution and stirred for 40 min. Thereafter 0.66 mL of aqueous solution containing $RE(NO_3)_3$ (0.2 M, $Y^{3+}/Yb^{3+}/Er^{3+}$ = 80/18/2 mol%) was added to the above solution and stirred for 1 h. The mixed solution was transferred to a Teflon-lined autoclave reactor, and reacted at 220 °C for 12 h. After the reaction was completed, it was naturally cooled down to room temperature, and the product was washed by ethanol and water for 3 times. Finally, the obtained white centrifugal product was dispersed into 2 mL of cyclohexane.

Synthesis of $NaYF_4$:Yb/Er hexagonal prisms

The $NaYF_4$:Yb/Er (18/2 mol%) hexagonal prisms were synthesized by a hydrothermal method. The synthesis procedure is as follows: 2.5 mL of ethanol, 2.5 mL of OA, and 7 mL of H_2O were added into 0.75 mL aqueous solution of NaOH (3.75 mmol) and the mixture was stirred for 40 min. Then 0.5 mL aqueous solution of NH_4F (2 M) was added into the above solution and stirred for 40 min. Thereafter 1 mL of aqueous solution containing $RE(NO_3)_3$ (0.2 M, $Y^{3+}/Yb^{3+}/Er^{3+}$ = 80/18/2 mol%) was added to the above solution and stirred for 1 h. The mixed solution was transferred to a Teflon-lined autoclave reactor, and reacted at 220 °C for 12 h. After the reaction was completed, it was naturally cooled down to room temperature, and the product was washed by ethanol and water for 3 times. Finally, the obtained white centrifugal product was dispersed into 2 mL of cyclohexane.

Synthesis of $NaYF_4$:Yb/Er/Gd nanorods

The $NaYF_4$:Yb/Er/Gd (18/2/30 mol%) nanorods were synthesized by a hydrothermal method. The synthesis procedure is as follows: 1.5 mL aqueous solution of NaOH (7.5

mmol) was added into 5 mL of ethanol and 5 mL of OA, and stirred for 40 min. Then an aqueous solution of NH_4F (2 M, 1 mL) and 2 mL of aqueous solution containing $RECl_3$ (0.2 M, Y^{3+}/Yb^{3+}/Er^{3+} /Gd^{3+} =50/18/2/30 mol%) was added into the above solution and stirred for 1 h. The mixed solution was transferred into a Teflon-lined autoclave reactor, heated at 200 °C for 2 h, and then naturally cooled down to room temperature. After completion of reaction, the product was washed three times with ethanol and water, and the obtained white centrifuged product was dispersed into 4 mL cyclohexane for future use.

Synthesis of $NaYF_4$:Yb/Er super microrods

The $NaYF_4$:Yb/Er (18/2 mol%) super microrods were synthesized by a hydrothermal method. The synthesis procedure is as follows: 2 mL of aqueous solution containing YCl_3 (0.4 mmol), $YbCl_3$ (0.09 mmol), $ErCl_3$ (0.01 mmol) was added into 1 mL of EDTA (0.5 mmol) aqueous solution and the mixture was stirred for 30 min. Then 6 mL aqueous solution of NaF (6 mmol) was added into the above solution and the pH of the solution was adjusted to 3 using HCl (1 M). The above mixture was stirred for 15 min. Then the mixture was transferred to a Teflon-lined autoclave reactor and reacted at 180 °C for 24 h. After the reaction was completed, it was naturally cooled down to room temperature. The product was collected by centrifugation and washed by ethanol and water for three times each. Finally, the obtained white product was dispersed into 5 mL of ethanol.

Fabrication of NIR-responsive photodetector

The NIR light photoresponse was tested on a film photodetector. Firstly, a square SiO_2/Si substrate with the edge length of 1 cm was cleaned by ethanol and acetone for

three times and dried at 50 °C in vacuum. Then 100 μL of ethanol solution containing MoS_2 sheets, MoS_2 tubes or $NaYF_4$:Yb/Er@MoS_2 composite with mass concentration of 2 mg/mL was drop-casted onto the surface of the chip. Then the chip was dried at 50 °C in vacuum. Au electrodes were deposited on the top of the chip via electron beam deposition through a shadow mask, defining photodetector channels with length of 100 μm and width of 1000 μm.

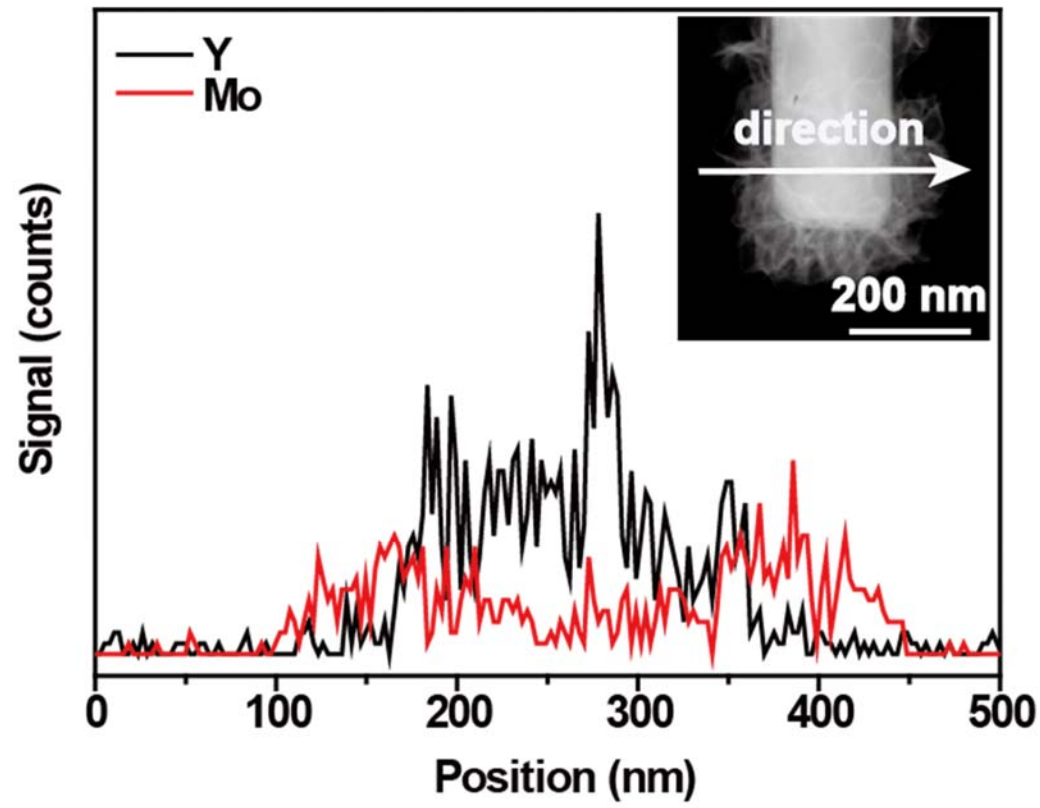

Figure S1. Line scan profiles of Y^{3+} and Mo^{4+} ions across a single rod of $NaYF_4$:Yb/Er@MoS_2 composite. The inset displays the sampling zone.

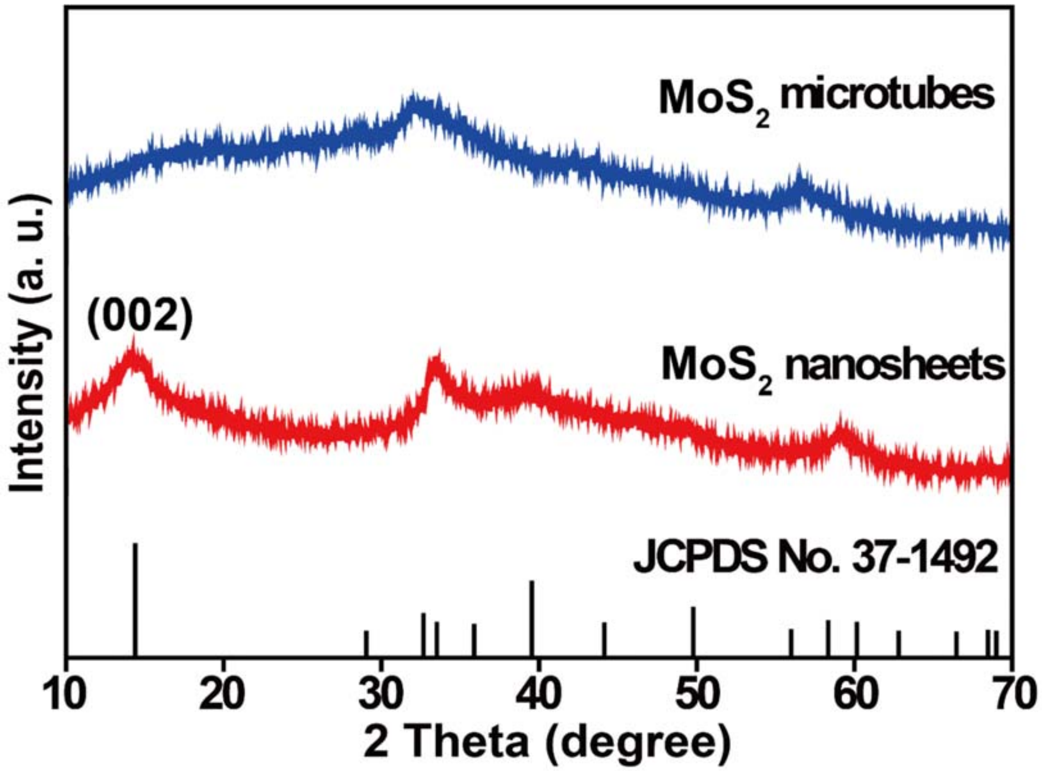

Figure S2. XRD patterns of MoS_2 nanosheets and MoS_2 microtubes. The pattern at bottom corresponds to the standard XRD data of hexagonal MoS_2 (JCPDS No. 37-1492).

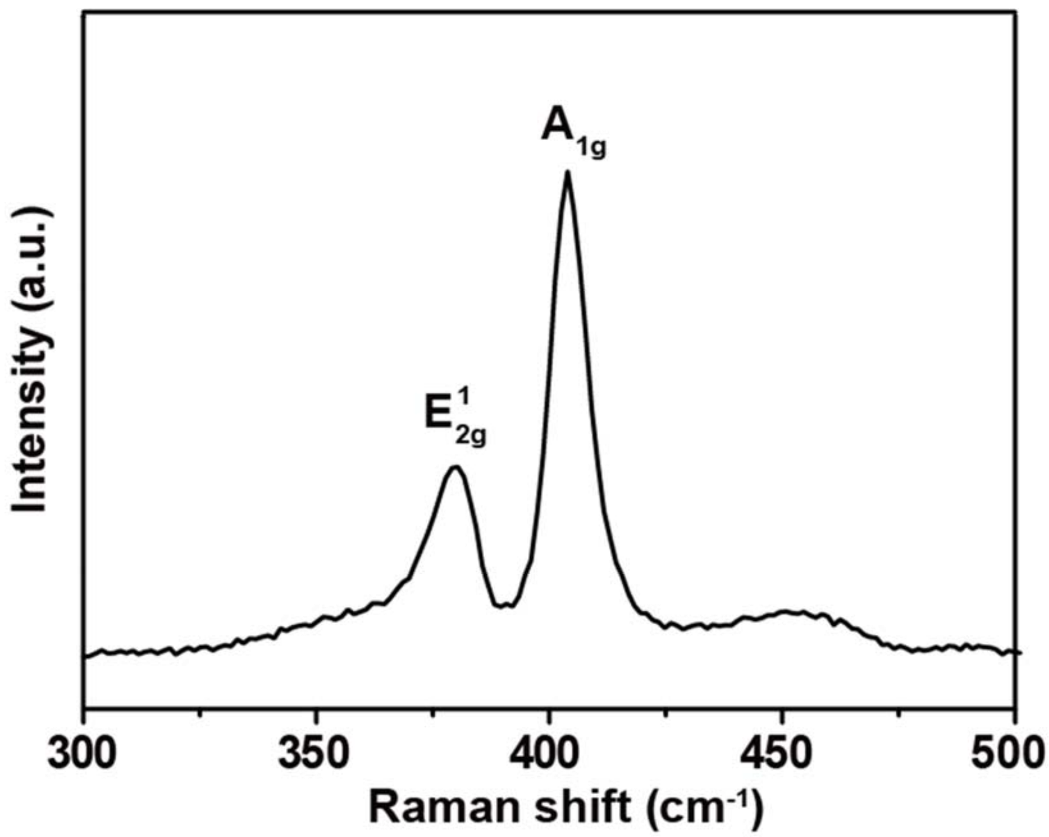

Figure S3. Raman spectrum of MoS_2 microtubes shown in Figure 1h.

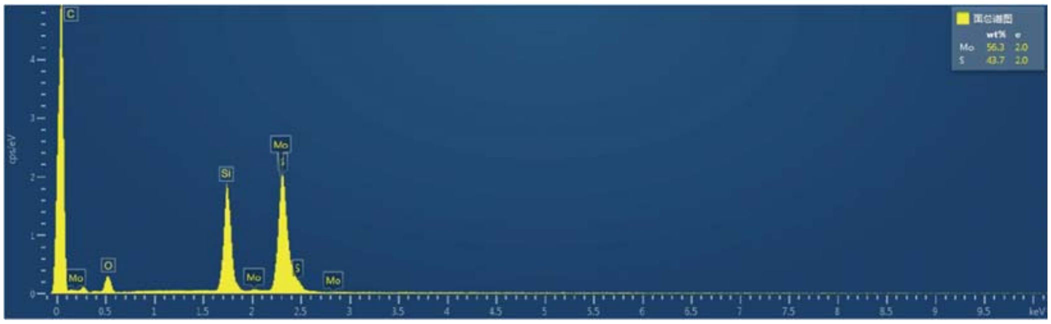

Figure S4. EDS analysis of hollow MoS_2 microtubes shown in Figure 1h.

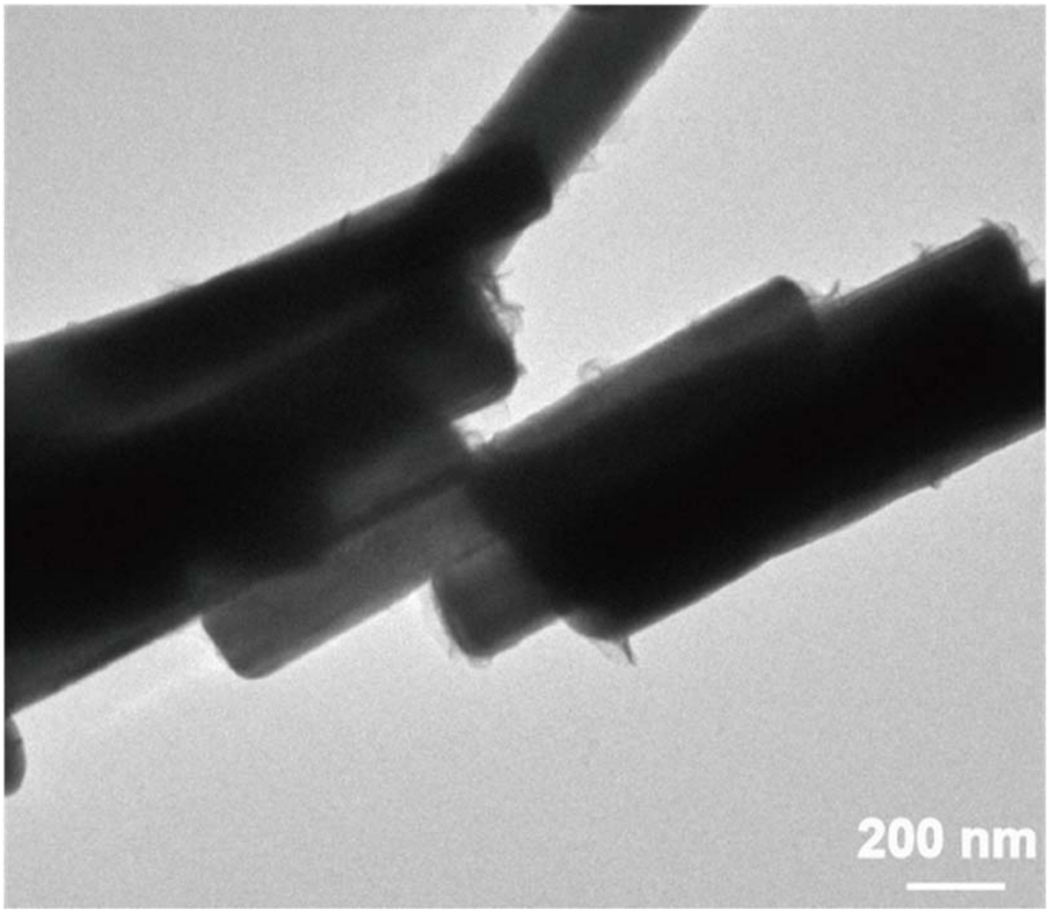

Figure S5. TEM image of $NaYF_4$:Yb/Er@MoS_2 composite prepared by hydrothermal reaction without using EDTA-2Na, where only very little MoS_2 nanosheets grew on the surface of $NaYF_4$:Yb/Er microrods.

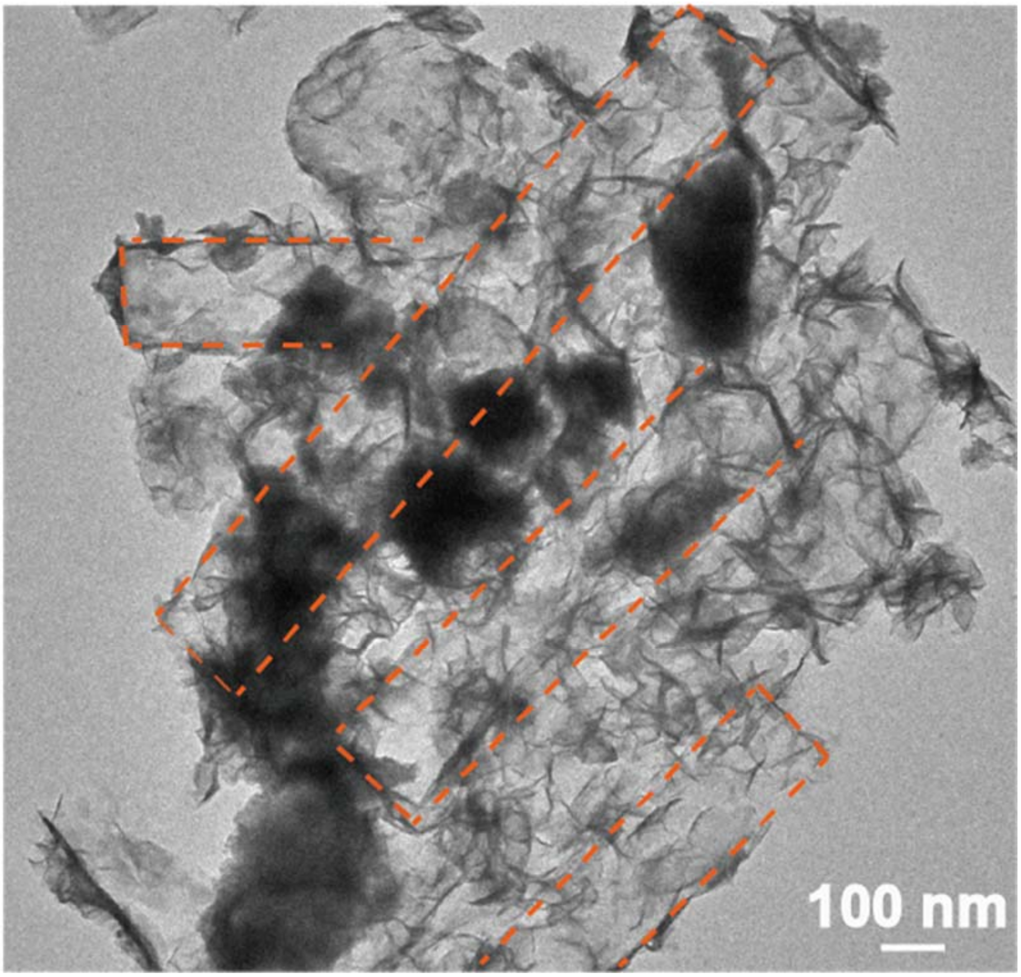

Figure S6. TEM image of the products obtained by etching off of the $NaYF_4$:Yb/Er template from $NaYF_4$:Yb/Er@MoS_2 composite synthesized without addition of EDTA-2Na, shown in Figure S5. The orange rectangular areas were supposed to be the microtubes of MoS_2, however only broken pieces were obtained due to the weak interaction between MoS_2 and $NaYF_4$:Yb/Er template.

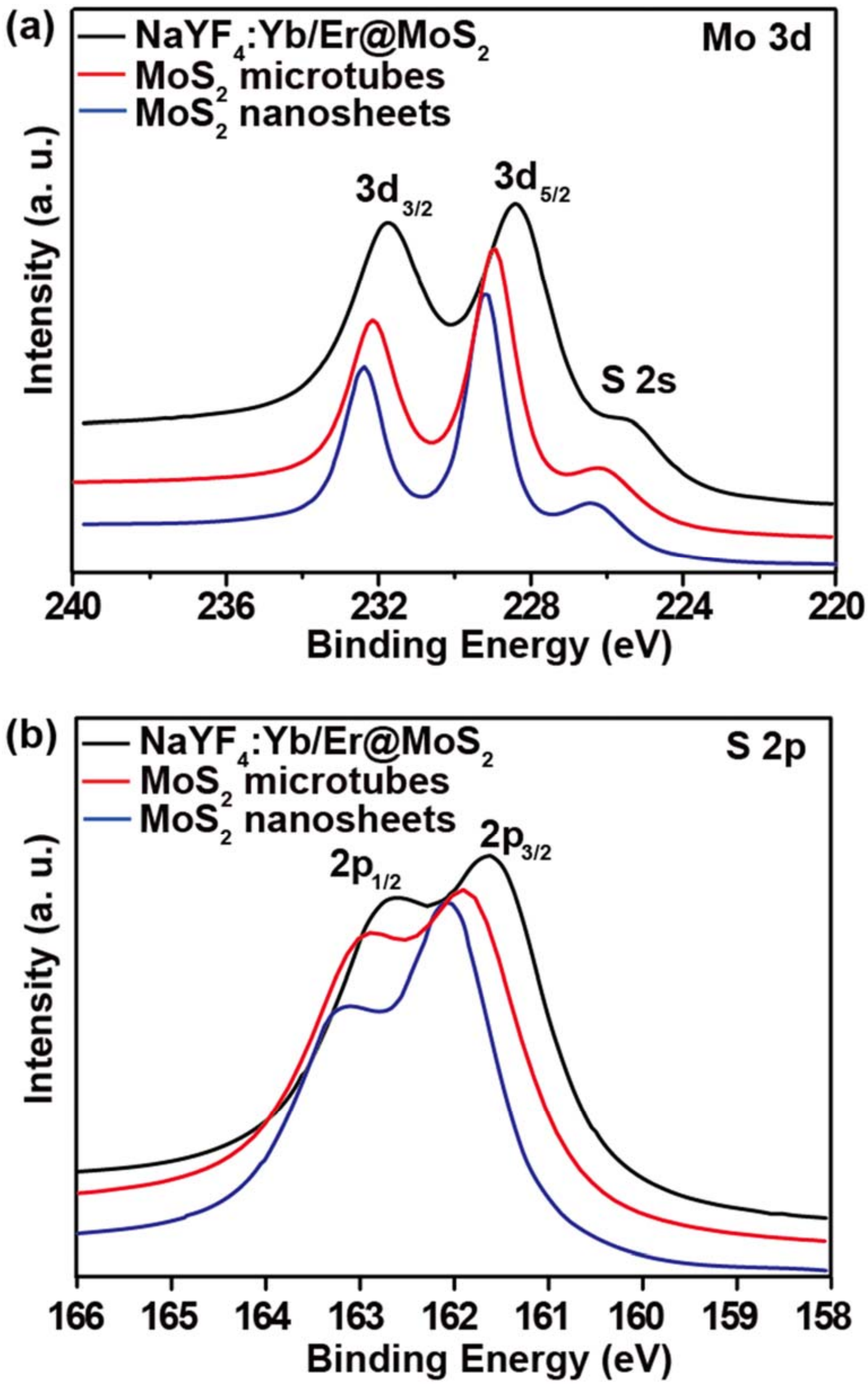

Figure S7. XPS showing the binding energies of (a) Mo 3d and (b) S 2p orbitals of MoS_2 nanosheets, MoS_2 microtubes, and $NaYF_4$:Yb/Er@MoS_2 composite.

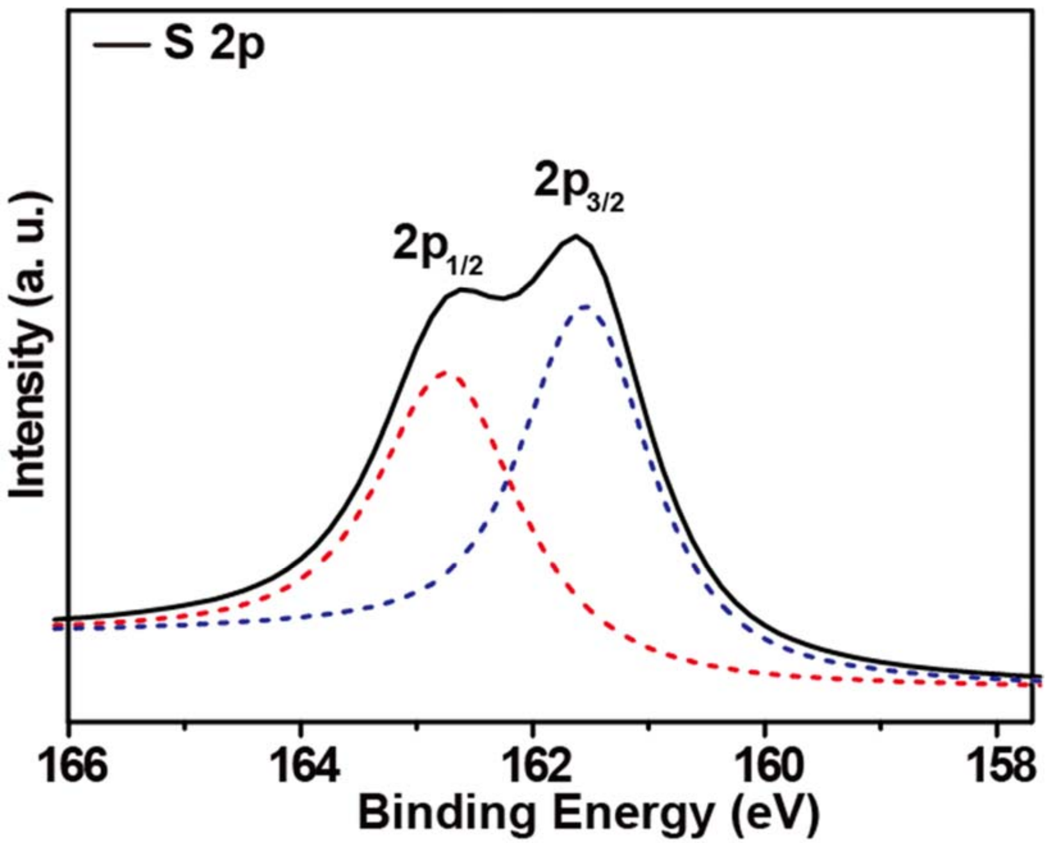

Figure S8. XPS showing the deconvoluted profiles of the binding energy of S 2p orbitals of $NaYF_4$:Yb/Er@MoS_2 composite.

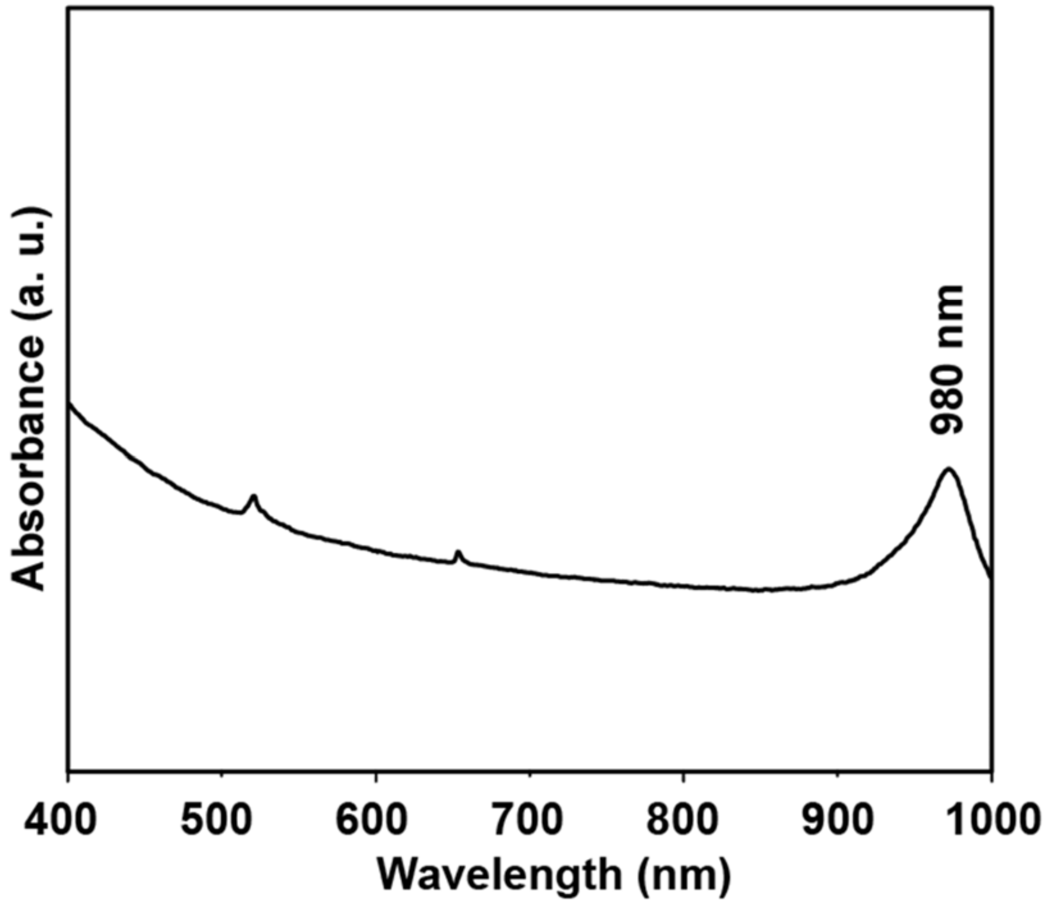

Figure S9. UV-Vis-NIR absorption spectrum of $NaYF_4$:Yb/Er microrods shown in Figure 1b.

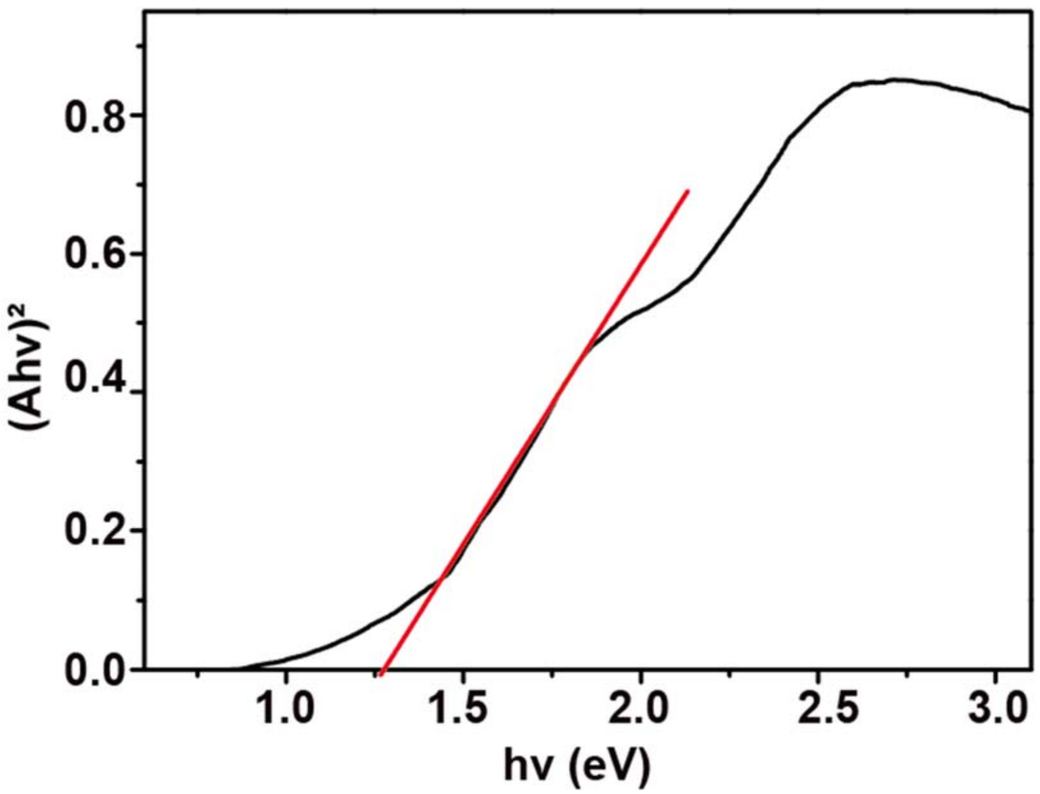

Figure S10. Tauc plot of as-synthesized MoS_2 thin layers demonstrating a bandgap of 1.276 eV.

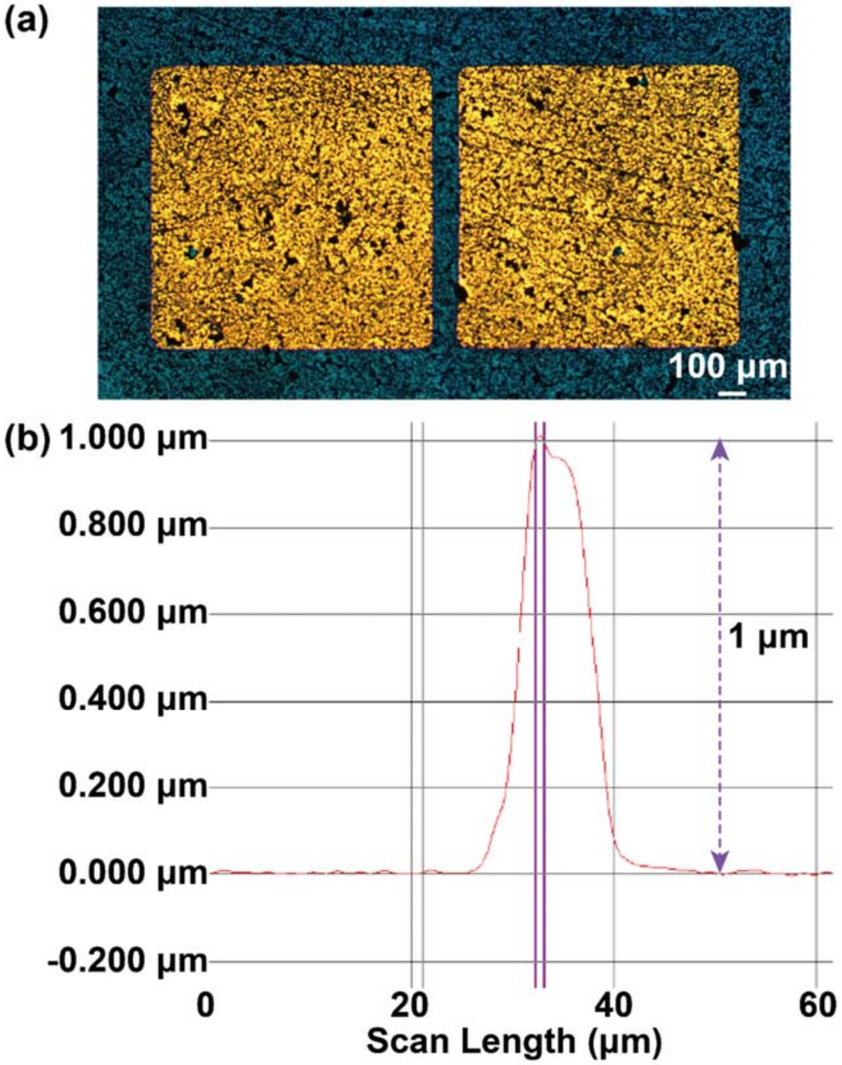

Figure S11. (a) Top-view optical image of the $NaYF_4$:Yb/Er@MoS_2 photodetector. The channel length and width are 100 and 1000 μm, respectively. (b) The measured thickness of $NaYF_4$:Yb/Er@MoS_2 film is about 1 μm through a step profiler.

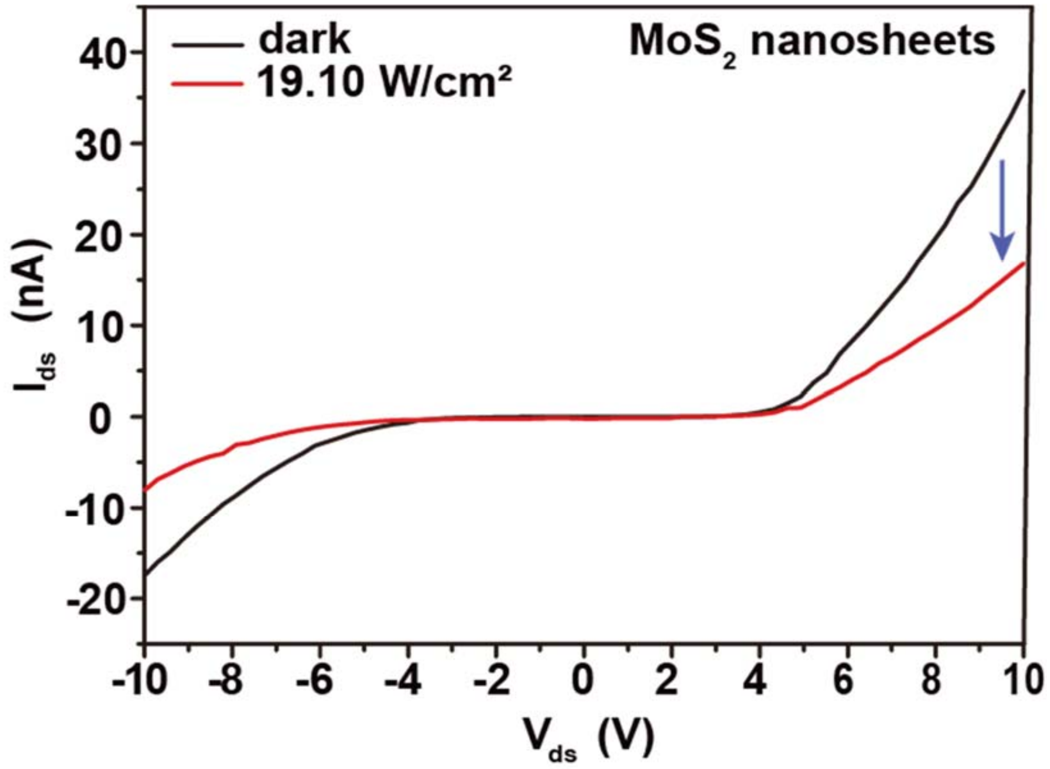

Figure S12. *I*-*V* curves of the photodetector made of MoS_2 nanosheets in dark and under 980 nm laser excitation.

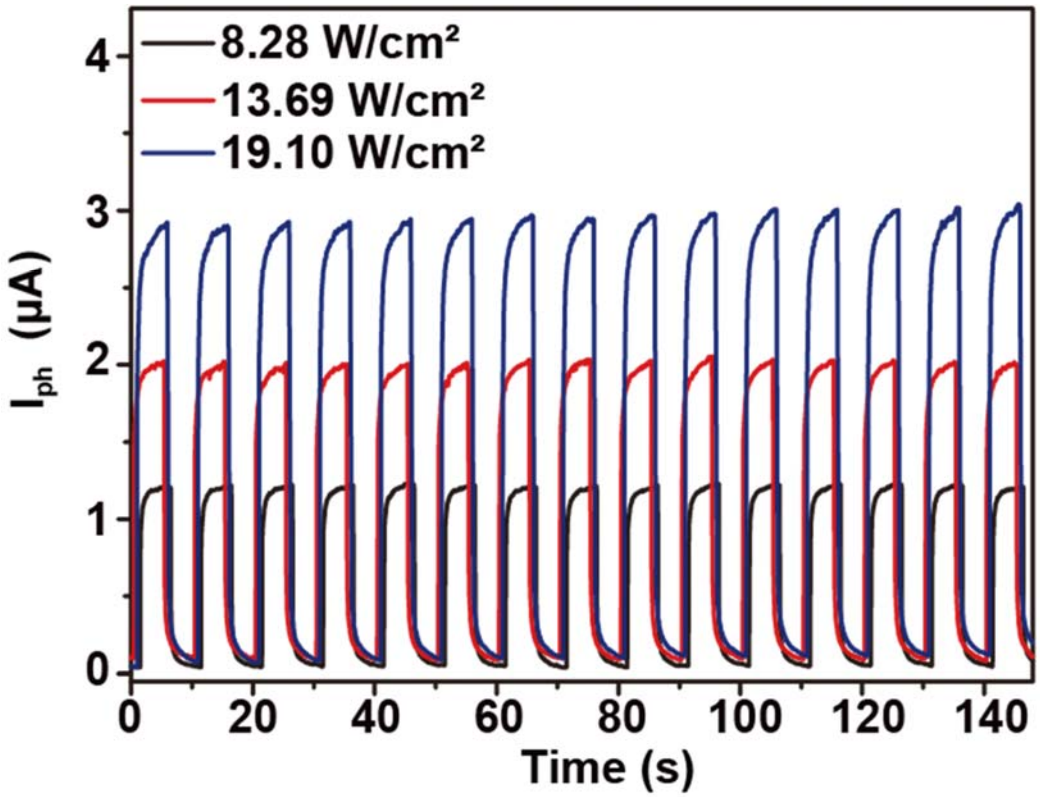

Figure S13. Temporal response of photodetector made of $NaYF_4$:Yb/Er@MoS_2 composite under 980 nm laser excitation (V = 10 V) at different power densities.

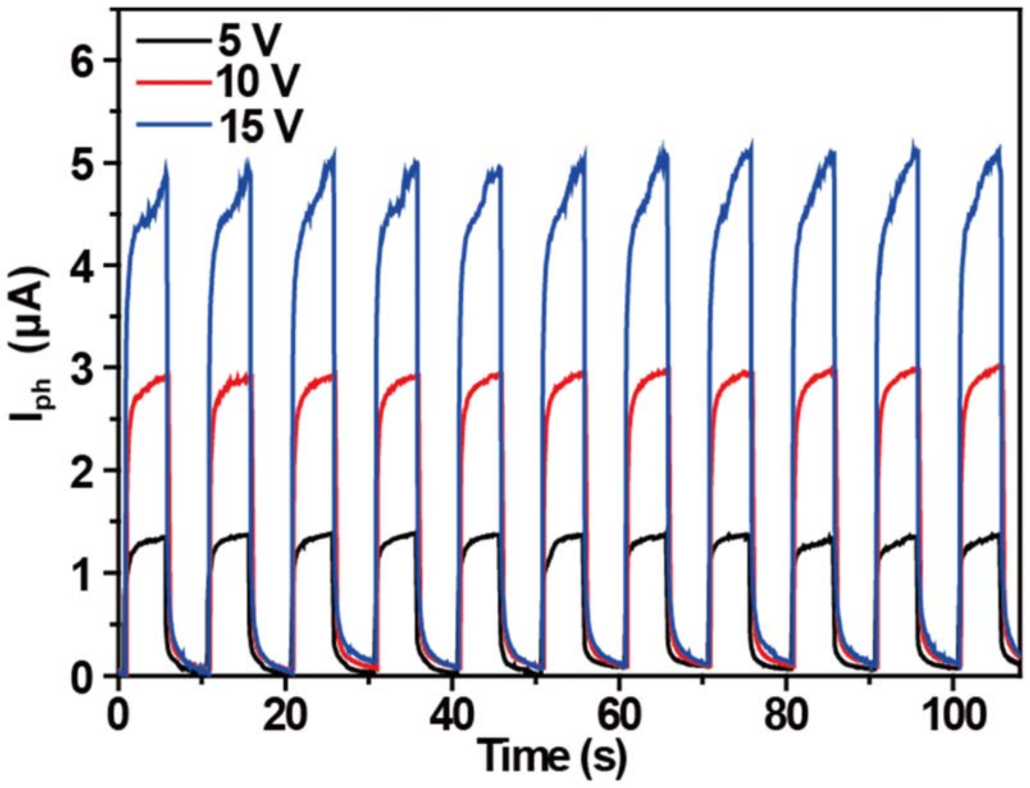

Figure S14. Temporal response of photodetector made of $NaYF_4$:Yb/Er@MoS_2 composite under 980 nm laser excitation at different voltages (power density 19.10 W/cm^2).

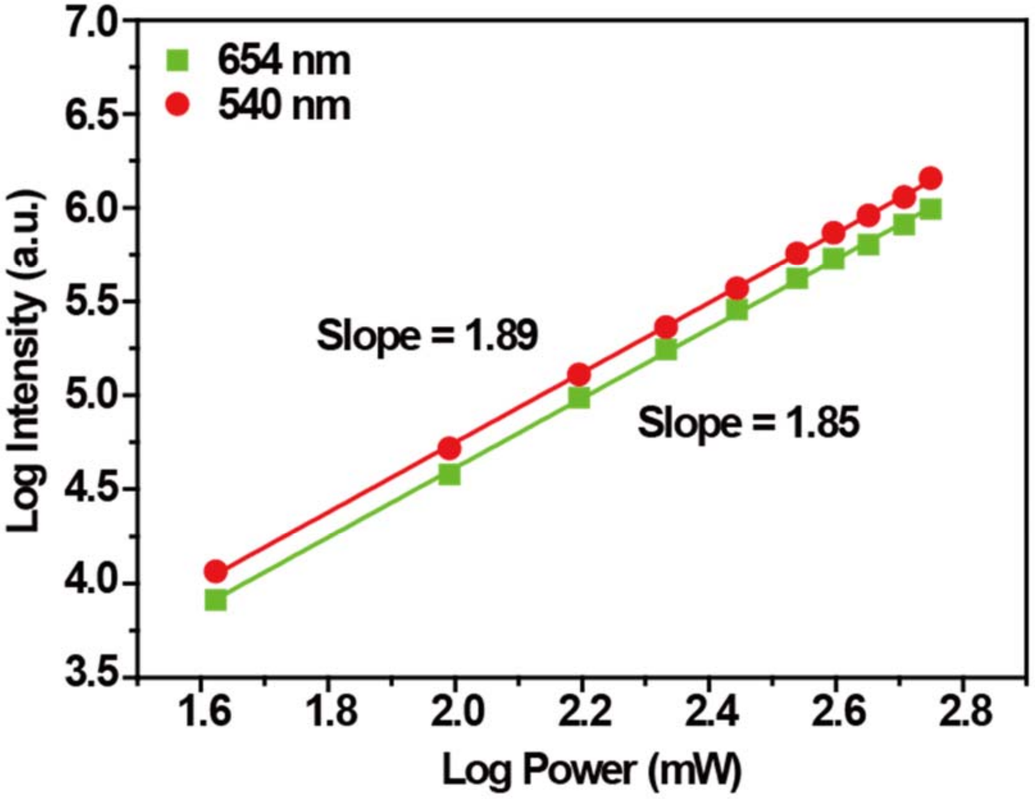

Figure S15. Log-Log curves of UCL emission intensities at 540 nm and 654 nm of $NaYF_4$:Yb/Er microrods *versus* 980 nm laser excitation power.

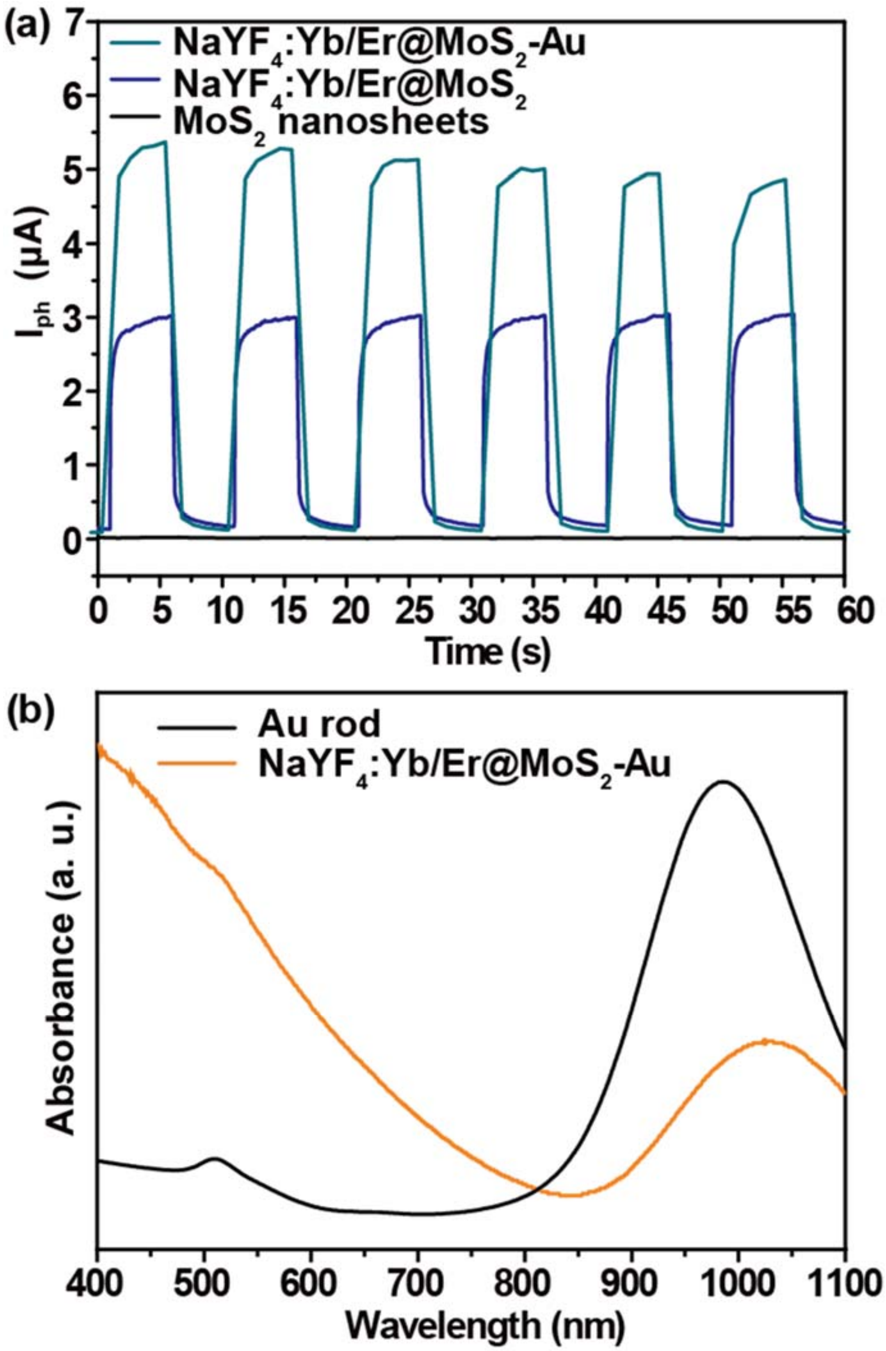

Figure S16. (a) Temporal photocurrent responsive characteristics of photodetectors made of $NaYF_4$:Yb/Er@MoS_2-Au composite, $NaYF_4$:Yb/Er@MoS_2 composite, and MoS_2 nanosheets , respectively, under 980 nm laser excitation (power density 19.10 W/cm^2, V = 10 V). (b) UV-vis spectra of Au nanorod and $NaYF_4$:Yb/Er@MoS_2-Au composite, showing overlapped absorption at 980 nm, the working wavelength of Yb^{3+}.

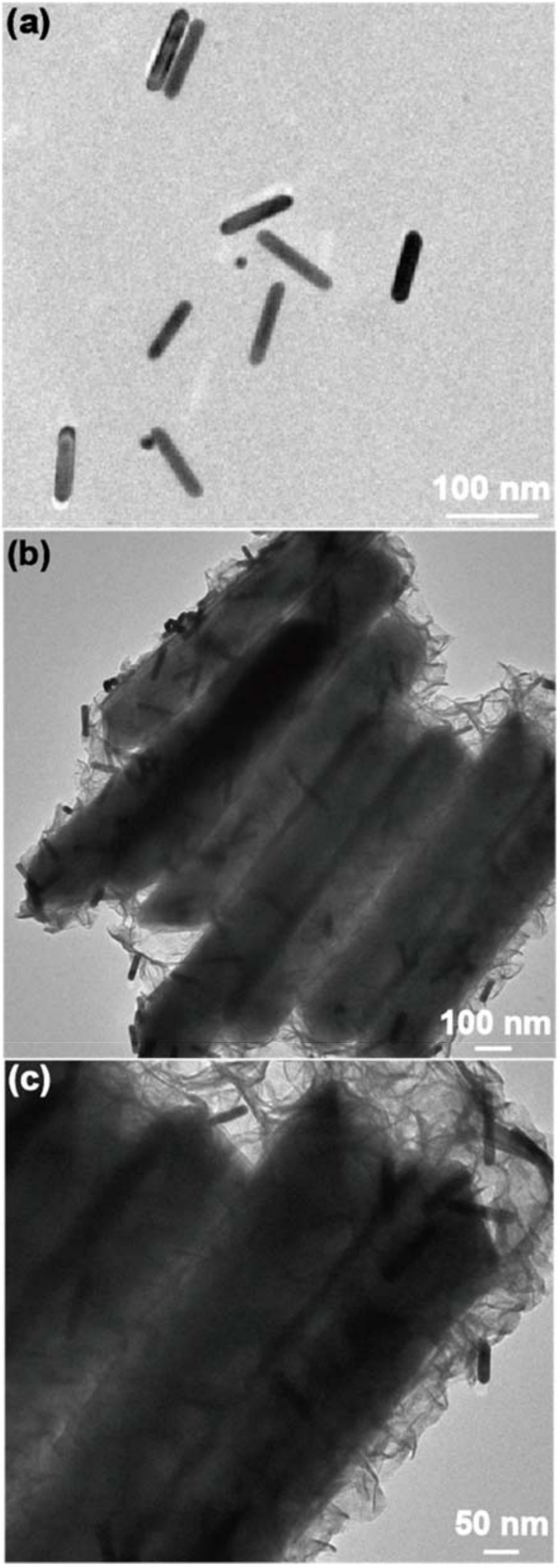

Figure S17. TEM images of (a) Au nanorods, (b) $NaYF_4$:Yb/Er@MoS_2-Au composite, and (c) zoomed-in TEM image of $NaYF_4$:Yb/Er@MoS_2-Au.

Table S1. Summary of the parameters of recently reported MoS_2 photodetectors.

Materials	Spectral range (nm)	Responsivity (mA/W)	Detectivity (Jones)	EQE (%)	Enhancement factor	Ref.
β-$NaYF_4$ UCMCs/MoS_2	633-1342	0.1 (980 nm)	10^8 (980 nm)	-	1000 times (980 nm)	[1]
PbSe-MoS_2	≥ 1200	-	-	-	~ 5 times	[2]
ZnS-MoS_2	365-780	0. 00452 (780 nm)	-	＜0.4 x 10^{-2} (780 nm)	-	[3]
$NaYF_4$:Ln @MoS_2	980/1532	0.166 (980 nm)	5.6 x 10^6 (980 nm)	2.1 x 10^{-2} (980 nm)	~ 600 times (980 nm)	This work

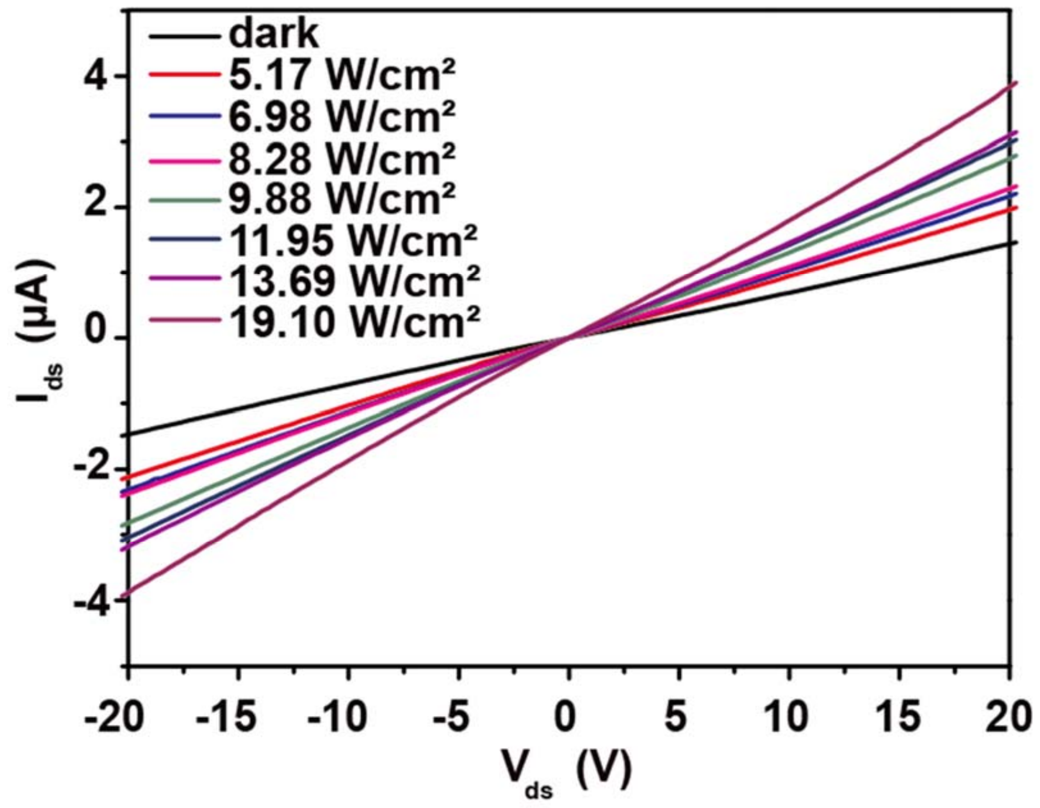

Figure S18. *I-V* curves of photodetector made of $NaYF_4$:Er@MoS_2 composite in dark and under 1532 nm laser excitation.

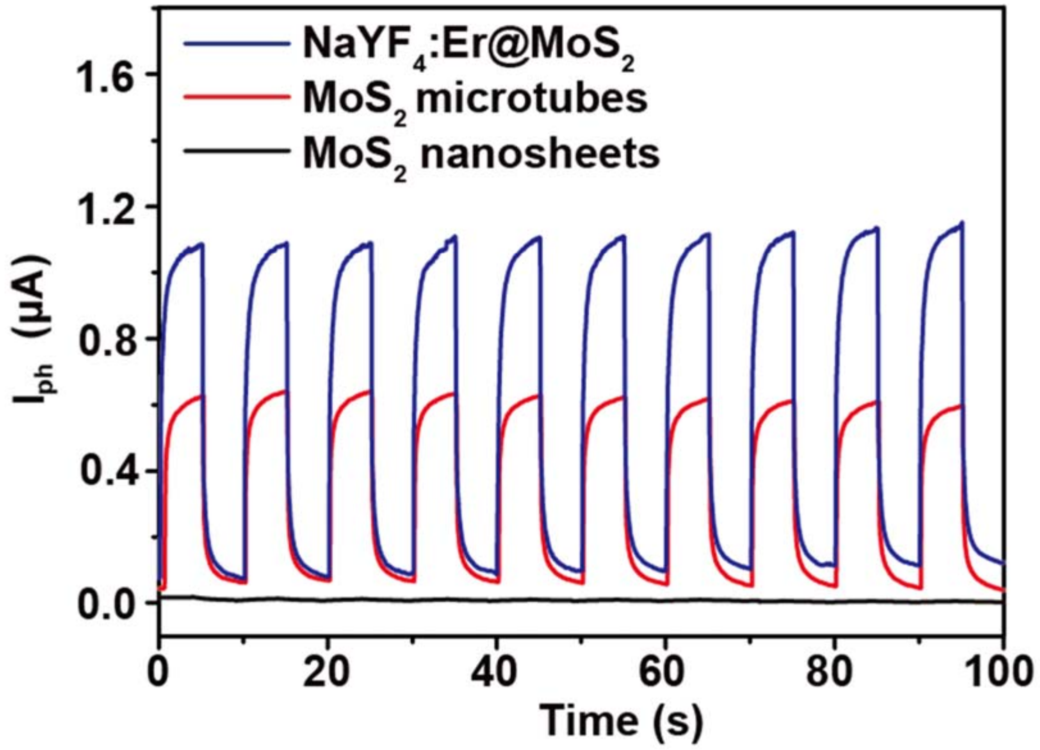

Figure S19. Temporal response of photodetectors made of MoS_2 nanosheets, MoS_2 microtubes, and $NaYF_4$:Er@MoS_2 composite, respectively, under 1532 nm laser excitation (power density 19.10 W/cm^2, V = 10 V). The photocurrents in devices made of MoS_2 microtubes and $NaYF_4$:Er@MoS_2 composites were enhanced by 155 and 276 folds, respectively, compared with that of MoS_2 nanosheets.

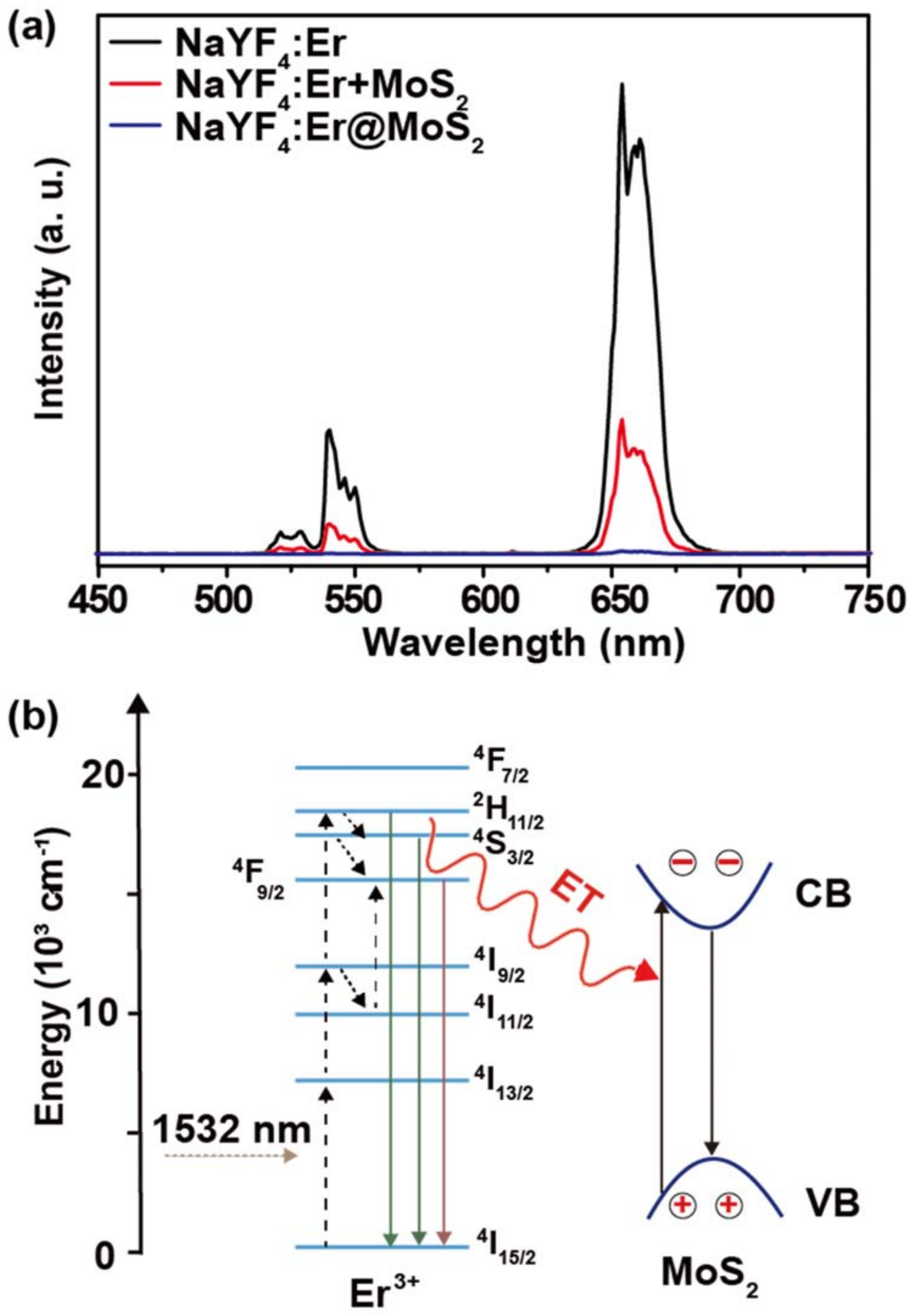

Figure S20. (a) Comparison of UCL of $NaYF_4$:Er microrods, $NaYF_4$:Er@MoS_2 composite, and physical mixture of $NaYF_4$:Er microrods and MoS_2 nanosheets under 1532 nm laser excitation. (b) Schematic diagram of energy transfer pathways from $NaYF_4$:Er to MoS_2 for generating electrons and holes under 1532 nm laser excitation.

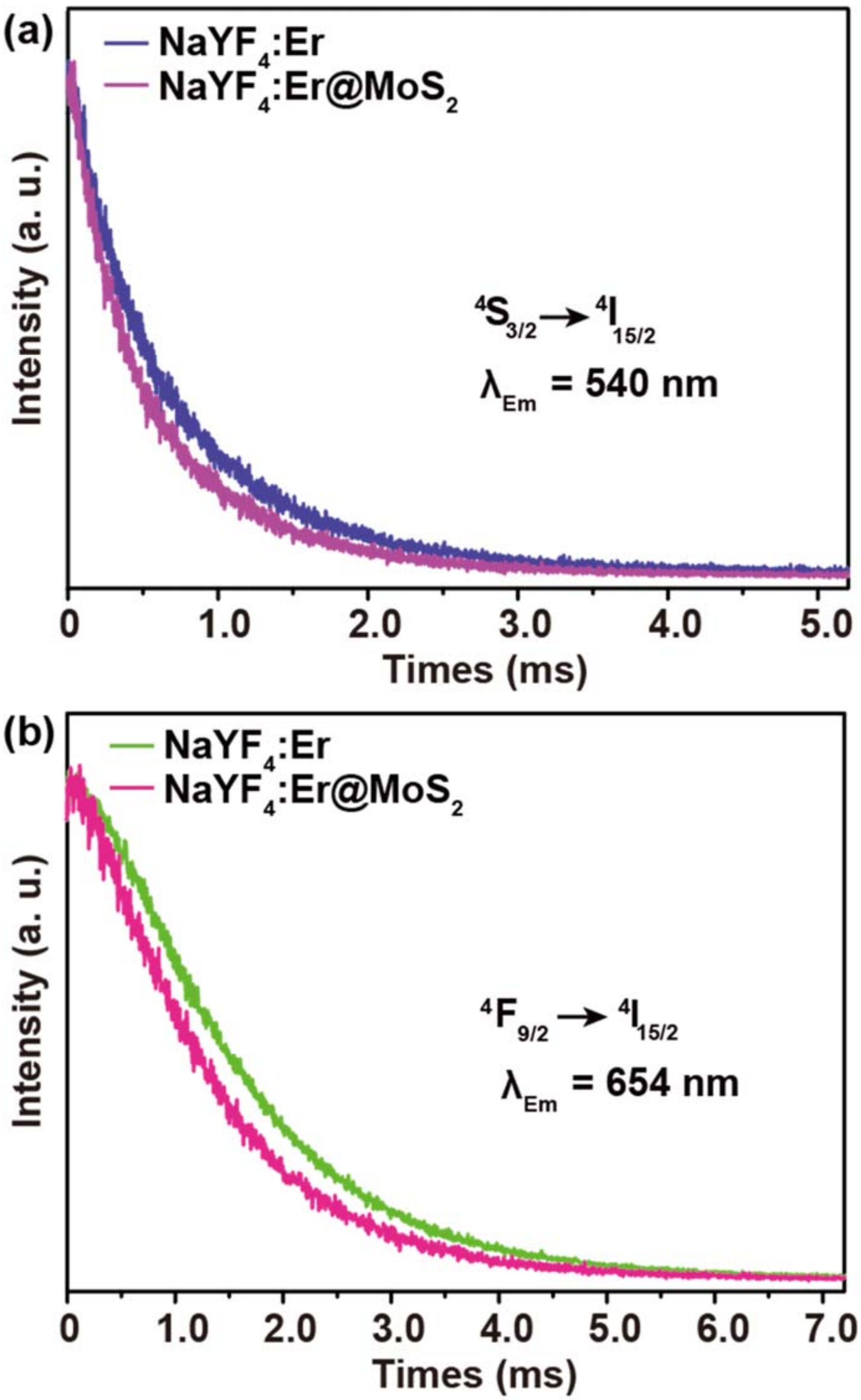

Figure S21. Luminescence decay curves of emissions at (a) 540 nm and (b) 654 nm in $NaYF_4$:Er microrods and $NaYF_4$:Er@MoS_2 composite, respectively, under 1532 nm laser excitation. (540 nm: 760 μs → 595 μs, 654 nm: 1.75 ms → 1.50 ms).

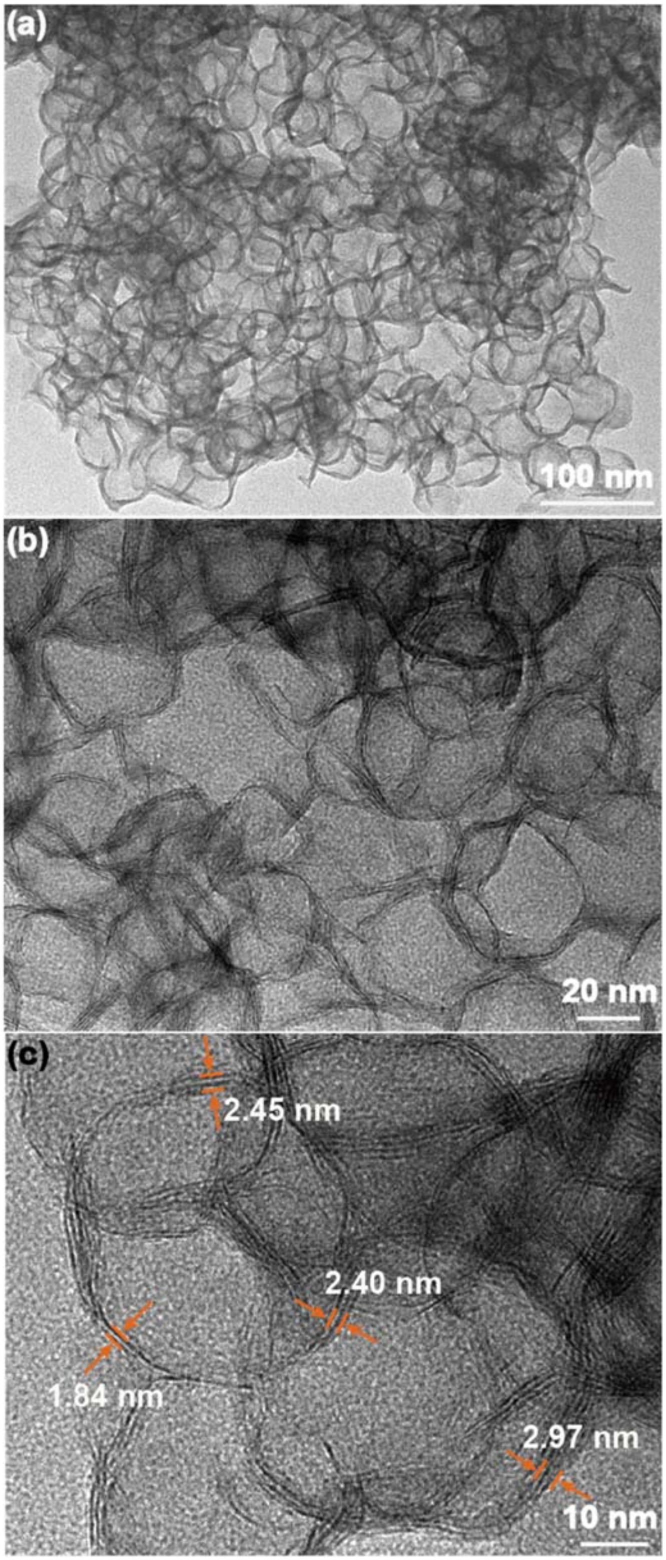

Figure S22. (a) TEM image of MoS_2 nanovesicles. (b) Magnified TEM image and (c) HRTEM image of sample in (a), showing the smallest thickness of the MoS_2 shell.

References

[1] Y. Zhang, J. Wang, B. Wang, J. Shao, J. Deng, C. Cong, L. Hu, P. Tian, R. Liu, S. L. Zhang, Z. J. Qiu, *Adv. Optical Mater*. **2018**, *6*, 1800660.

[2] J. Schornbaum, B. Winter, S. P. Schießl, F. Gannott, G. Katsukis, D. M. Guldi, E. Spiecker, J. Zaumseil, *Adv. Funct. Mater*. **2014**, *24*, 5798.

[3] P. T. Gomathi, P. Sahatiya, S. Badhulika, *Adv. Funct. Mater*. **2017**, *27*, 1701611.
